# Graphene‐Based Material Supports for Ni− and Ru− Catalysts in CO_2_ Hydrogenation: Ruling out Performances and Impurity Role

**DOI:** 10.1002/cssc.202400993

**Published:** 2024-10-10

**Authors:** Sina Ebrahim Atakoohi, Paola Riani, Elena Spennati, Letizia Savio, Luca Vattuone, Jacopo De Maron, Gabriella Garbarino

**Affiliations:** ^1^ Department of Civil, Chemical, and Environmental Engineering University of Genova Via Opera Pia 15 16145 Genova Italy; ^2^ Department of Chemistry and Industrial Chemistry University of Genova Via Dodecaneso 31 16146 Genova Italy; ^3^ INSTM, UdR Genova Via Dodecaneso 31 16146 Genova Italy; ^4^ IMEM-CNR Via Dodecaneso 33 16146 Genova Italy; ^5^ Department of Physics University of Genova Via Dodecaneso 33 16146 Genova Italy; ^6^ Department of Industrial Chemistry University of Bologna Viale del Risorgimento 4 40126 Bologna Italy

**Keywords:** Carbon-based, CO_2_hydrogenation, Heterogeneous catalysis, Molten salt, rWGS, Supported catalysts

## Abstract

Laboratory‐prepared Gnp using molten salt, commercial Gnp and reduced graphene oxide (rGO) have been characterized and utilized as support for CO_2_ hydrogenation catalysts. Ni− and Ru− catalysts supported over Gnp, commercial Gnp and rGO have been deeply characterized at different stages using Raman, IR, XRD, FE‐SEM‐EDXS, SEM‐EDXS, XPS, and TEM, also addressing carbon loss before reaction and evolved species, thus allowing a better comprehension of the produced materials. Ni and Ru/rGO were inactive while Gnp‐supported ones were active. Ru has been found almost completely selective toward reverse Water Gas Shift to CO, approaching the forecasted thermodynamic equilibrium at 723 K, in the tested conditions (Y_CO_~55 %), with an apparent activation energy in the range of 70–90 kJ/mol. Exhaust catalysts pointed out the presence of sulfur partially linked to the carbon matrix and partially producing the corresponding metal sulfide with the detection of surface oxidized species in the cationic form and adsorbed species as well. The metal‐based nanoparticles displayed a quite narrow size distribution, confirming the promising behavior of these catalytic systems for CO_2_ utilization.

## Graphical abstract

Ni and Ru catalysts, supported over laboratory‐made Gnp, commercial Gnp, and rGO, are prepared characterized, and tested in CO_2_ hydrogenation, ruling out the role of impurities. Ru‐Gnp exhibits high selectivity, among tested materials, for the reverse Water Gas Shift reaction, approaching thermodynamic equilibrium at 723 K, while rGO‐based catalysts remain inactive.

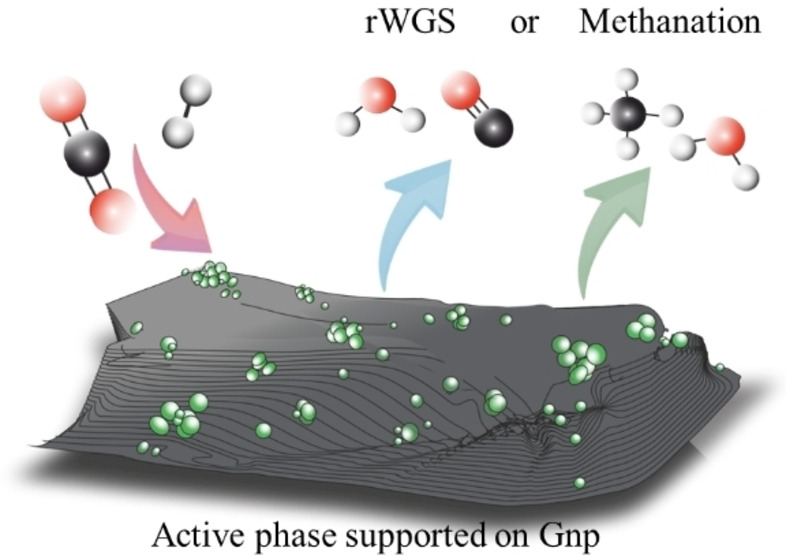

## 1 Introduction

Over the recent years, attention has increasingly turned towards the hydrogenation of captured CO_2_ using green hydrogen to produce e‐fuels in the frame of Power to X technologies (PtX).[^1^, ^2^] These approaches show potential in reducing greenhouse gas emissions and promoting a sustainable energy cycle.[^3^, ^4^, ^5^, ^6^, ^7^, ^8^] Potential routes for the valorization of CO_2_ involve its conversion into carbon monoxide, methane, methanol, formic acid, synthetic fuels, olefins, etc.^[9]^ In this frame, it is useful to remark that CO production, through reverse Water Gas Shift (rWGS) can be used in the two‐stage syntheses in the case of methanol, Fischer Tropsch, and high alcohol, achieving higher conversion and selectivities. Main mentioned reactions are[^10^, ^11^, ^12^]:
(1)

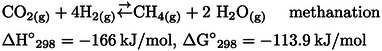



(2)

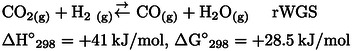



(3)

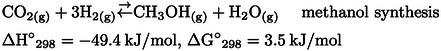



(4)

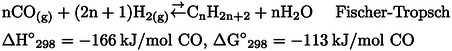



(5)

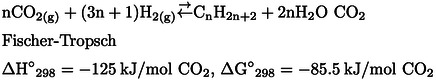



(6)

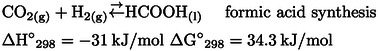




Suitable process conditions need to be applied considering the quite complex reaction network that could also include side reactions, that might poison the catalysts or reduce the selectivity, as in the case of methanation where rWGS is an unwanted side reaction.

Thus, the development of active and selective catalysts for CO_2_ hydrogenation could significantly contribute to enhancing process efficiency as recently reported.^[13]^

Among the diverse approaches to CO_2_ utilization, this study aims at the conversion of CO_2_ to Synthetic Natural Gas (SNG) and carbon monoxide (CO) through the CO_2_ methanation reaction (Equation (1)) and the reverse water‐gas shift (rWGS) reaction (Equation (2)), respectively. As a function of the operational conditions i. e., temperature and partial pressures, several reactions might compete with each other^[14]^ and, in some cases, also C deposition[^15^, ^16^, ^17^] need to be accounted:
(7)

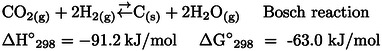



(8)

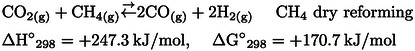



(9)

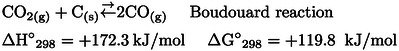




Among suitable active phases, transition metals are generally employed and often good performances in terms of conversion are reported. In fact, conventional catalysts are primarily based on rare metals, noble metals, transition metals, metal oxides, or hybrids that might face limitations due to high costs, toxicity, and environmental concerns.[^18^, ^19^] In particular, in the case of CO_2_ methanation reaction, also known as the Sabatier reaction, those based on Ru[^8^, ^20^, ^21^, ^22^] and Ni[^4^, ^5^, ^7^, ^23^, ^24^, ^25^] have shown significant potential. On the other side, when dealing with CO_2_ to CO conversion (rWGS), the interest is primarily focused on Fe‐based catalysts,^[26]^ although there are also efforts to explore Ni‐based catalysts.^[27]^ Also, platinum group metals are exploited together with several bimetallic formulations, but the poor availability and high cost are the main bottlenecks for their use accounting for specific activity, Ru>Fe>Ni>Co>Rh>Pd>Pt>Ir and for selectivity and activity trends: Ni>Co>Fe>Ru and Ru>Fe>Ni>Co, respectively. Nevertheless, there has been an exploration into noble metals (Pt,[^28^, ^29^] Pd,^[30]^ Ru,^[31]^ Rh,^[32]^ Au^[33]^) and non‐precious transition metals (Fe,[^26^, ^34^] Ni,^[35]^ Co^[36]^) supported on different metal oxides.

Ni‐based catalysts are frequently employed for both rWGS and methanation reactions because of their high catalytic activity, acceptable robustness, and low cost.[^37^, ^38^, ^39^, ^40^, ^41^] Moreover, various metal oxides have been used as catalyst support for both reactions, including Al_2_O_3_, CeO_2_, SiO_2_, and ZrO_2_, TiO_2_.[^26^, ^42^, ^43^] A non‐negligible role is played by structural and chemical promoters that allow to enlarge temperature operation window and enhance selectivity toward the wanted products; among others, both lanthanides^[44]^ i. e., La, Ce, Pr, Gd, and *d‐group* metals have been investigated[^7^, ^45^, ^46^, ^47^] As well, the support can play a significant role in CO_2_ activation and appropriate hydrogenation,^[48]^ Together with the promoters, literature works indicate that tuning Strong Metal‐Support Interaction (SMSI), the size of the active metal, and the loading of an eventual second metal, can influence the selectivity toward either CO or CH_4_.

Deactivation can also occur through different pathways, including coking, sintering, poisoning, and structural change that might happen in exothermal processes, with the subsequent reduction of catalysts’ lifetime. As an example, sintering often results in the loss of accessible active sites, leading to a decline in catalytic activity, and, in Ni− case, extended particles could also be involved in carbon encapsulation.[^49^, ^50^] Therefore, over recent years, different catalytic materials have been explored as potential alternatives, balancing activity, abundance, efficiency, robustness, sustainability, and cost.[^18^, ^19^, ^42^, ^51^, ^52^]

In the search for alternatives, carbon‐based materials are desirable for catalyst support applications due to their natural abundance, low cost, superior stability in both acidic and base conditions, and high catalytic activity.[^53^, ^54^, ^55^, ^56^, ^57^] Moreover, the use of doped graphene is achieving great interest for several applications. Recent reports suggest that the use of sulfur‐doped graphene can enhance the anchoring of Au_25_ catalyst for nitrogen reduction reaction, displaying a quite stable behavior.^[58]^ Also, a recent attempt to produce nickel sulfides, supported on N‐ and S‐doped graphitic carbon nitride^[59]^ for sensing, and on graphene for Li‐ion batteries, has been proposed.^[60]^ Moreover, it is important to underline that the use of external nonmetal heteroatoms i. e., nitrogen, phosphorus, sulfur doping, provides the capacity to tailor the charge redistribution, CO_2_ affinity, and electrical conductivity, producing more active catalysts in eCO_2_RR (electrochemical CO_2_ reduction reaction).^[61]^ Thus, the purity of starting materials and the preparation route might introduce heteroatoms that could strongly affect catalytic activity, acting as a poison or sometimes even as an anchoring site. Furthermore, among parameters that can play a role, the thermal treatment could affect the final catalyst. The role of C‐based materials in CO_2_ hydrogenation has also been recently reviewed,^[9]^ and the interest in the topic is rapidly growing, also by looking at hybrid catalytic systems that efficiently couple a conventional catalyst with reduced Graphene Oxide (rGO), Quantum Dots, graphene, etc.^[62]^

Wu et al. have demonstrated that the structure of carbon‐based materials can be modified to enhance their activity for CO_2_ hydrogenation^[63]^ showing, in particular, that the activity is ruled by the dimension and defect density of the carbon nanomaterials: reducing the dimension of graphene down to graphene quantum dots (GQD, lateral size of <5 nm) and further introduction of nitrogen (N) dopants greatly promotes the CO_2_ hydrogenation activity.

A quite wide variety of carbon materials in 1D, 2D and 3D structures are available and are under investigation for their unique properties, even though several challenges are still open. Among others, the synthesis methods for monolayer or a few‐layer graphene encounter significant challenges, characterized by drawbacks such as the use of harsh chemicals, high energy requirements, expensive equipment, low yield, poor dispersion, prolonged processes, and potential damage to the final product. These issues collectively hinder the comprehensive commercialization of graphene.^[64]^ Due to these considerations, innovative attempts have been made to utilize easily producible 3D graphene‐like structures such as graphene aerogels or porous graphene. These materials combine the benefits of the 2D graphene structure in their walls with the macro and microporosity, as well as the high surface area achieved by the 3D structure.^[64]^ One of the most innovative approaches for synthesizing porous graphene is the Molten Salt (MS) exfoliation strategy, employing pristine graphite as a precursor.^[65]^ In this approach, the melt of salts such as LiCl, KCl, NaCl, ZnCl_2_, MgCl_2_, CaCl_2_, CsCl, LiOH, KOH, and KNO_3_ or a eutectic mixture of them can be used as exfoliative media to separate layers of graphene from pristine graphite. This method offers advantages such as simplicity without the requirement for complex devices, good efficiency, scalability, low cost, environmental friendliness, and sustainability, even though limited catalyst applications and investigation on impurity roles are available.[^65^, ^66^, ^67^, ^68^, ^69^, ^70^, ^71^, ^72^, ^73^, ^74^, ^75^]

In this study, the MS method was employed to synthesize a graphene‐like material known as graphene nanoplatelet (henceforth referred to as Gnp), consisting of a few stacked layers of graphene. Subsequently, this Gnp, produced in the laboratory, was utilized as the catalyst support for CO_2_ hydrogenation. Furthermore, commercial Gnp and rGO were also employed as reference catalyst supports, and results in terms of performance were compared, by ruling out the role of sulfur, which can either be present in the starting material or introduced in trace amounts during catalyst preparation.

## Materials and Methods

### Materials

For the preparation of investigated catalysts, the following materials have been used: graphite powder (Acros Organics, ~99.0 % purity), graphene nanoplatelet (henceforth referred to as Com.Gnp; Nanografi, 99.9 % purity), reduced Graphene Oxide (rGO; Nanografi, 99 % purity), sodium chloride (Carlo Erba, assay 99.0–100.5 %, calculated on dried substance), potassium chloride (Carlo Erba, assay 99.0–100.5 %, calculated on dried substance), nickel(II) nitrate hexahydrate (Alfa Aesar, ~98.5 %), ruthenium(III) nitrosyl nitrate (Alfa Aesar, 1.5 % w/v Ru), ethanol (Carlo Erba, absolute anhydrous >99.8 %).

### Synthesis of Gnp

The molten salt method was utilized to synthesize Gnp, following an optimized synthetic route developed from the one reported by Ruse et al.^[76]^ In this method, a mixture of molten NaCl‐KCl was used to exfoliate graphite to Gnp. Accounting for the hygroscopic properties of NaCl and KCl, both salts were initially treated at 773 K for 3 hours. Once cooled to 423 K, they were transferred to a vacuum drier and stored until use. To prepare the graphite‐salt composite, a mixture of NaCl and KCl (molar ratio=1) was mixed with graphite powder (with a graphite‐salt weight ratio of 1 : 9) in a mortar to create a homogeneous mixture. This was subsequently placed into an alumina crucible, sealed in an Ar‐filled quartz tube, heated up to 1023 K (higher than the NaCl‐KCl azeotropic temperature^[77]^) at a rate of 10 K/min, and held at 1023 K for 360 minutes. After cooling to room temperature, the produced graphite‐salt composite was collected and characterized. To separate Gnp from other C‐based materials, the graphite‐salt composite was dispersed in ethanol^[76]^ and sonicated for 2 hours in an ice bath. Afterward, the resulting dispersion was settled for 20 minutes, and the supernatant was collected and filtered (Sartorius pore diameter 0.2 mm). The obtained material was washed with hot water several times, filtered, and finally dried at 353 K for 24 hours. The notation adopted for this C‐based material was Gnp. The whole synthesis allowed us to reach a Gnp yield of ~33 wt %, in line with the literature work.^[72]^

### Catalyst Preparation

Gnp, commercial Gnp (Com.Gnp), and rGO were used as support for the investigated catalysts. All the materials were prepared by incipient wetness impregnation method using ethanol as solvent and Ni(NO_3_)_2_*6H_2_O, and Ru(NO)(NO_3_)_3_ as metal precursors to obtain the following catalysts: 2 %Ni/Gnp, 2 %Ni/Com.Gnp, 2 %Ni/rGO, 0.5 %Ru/Gnp, 0.5 %Ru/Com.Gnp, and 0.5 %Ru/rGO, where the reported metal percentage is always in wt.%. Each catalyst underwent a two‐step in‐situ drying‐reducing process before the catalytic test. Each material was heated to 773 K at a rate of 10 K min^−1^ in a flow of N_2_ and kept for 30 min in the same atmosphere then changed to 20 mol % H_2_–80 mol % N_2,_ maintained for another 30 minutes, before natural cooling down. Throughout the process just described, the flow rate remained consistently stable at 80 NmL min^−1^. The Gnp, prepared with molten salt, was also tested without any active phase loading, and designated as Gnp/Metal‐Free.

### Catalytic Experiments

In line with our previous studies,[^6^, ^23^] catalytic experiments were conducted using a tubular silica glass flow reactor containing a fixed bed filled with 88.0 mg of catalyst mixed with 700 mg of silica glass beads (0.25–0.21 mm, equivalent to 60–70 mesh sieved). Before the catalytic activity, the catalysts were *in situ* reduced using the procedures mentioned earlier. CO_2_ hydrogenation experiments were performed using 80 NmL min^−1^ of the following feed gas composition: 6 % CO_2_, 30 % H_2_, balanced with N_2_ as the carrier gas. The gas hourly space velocity (GHSV) was set at 4850 h^−1^, considering the total bed volume V_tot_, consisting of silica glass and catalyst powder. To investigate potential hysteresis, activation, or short‐term deactivation effects, the experiments were conducted in both ascending and descending temperature sequences (503 K–723 K, and vice versa with a 50 K step) and a temperature gradient was simulated in the furnace by reducing the temperature of 70 K along the length axis, in line with the presence in concentrated conditions of a hot‐spot at the bed entrance.[^78^, ^79^] In this case, the average temperature of the catalytic bed will be used for the comparison of performances, accounting for both catalyst and stream dilutions.[^80^, ^81^] Moreover, as a reference, one of the catalysts has been tested in the isothermal regime, for comparison purposes (dashed lines). The complete experiment lasted for 7 hours under continuous operation.

To perform online product analysis, a Nicolet 6700 FT‐IR instrument was employed. Frequencies corresponding to the weak absorption of CO_2_, CH_4_, and CO molecules were utilized (namely, 2293 cm^−1^ for CO_2_, 2170 cm^−1^ for CO, and 1333 cm^−1^ for CH_4_, after baseline subtraction). The instrument underwent calibration using gas mixtures with known concentrations to guarantee precise and quantitative results throughout the analysis. Most of the produced water was condensed upstream of the IR cell. By considering the inlet and outlet concentrations calculated using the absorbances of CO_2_, CH_4_, and CO, along with the measured total flows to account for variations in moles during the reaction, parameters such as CO_2_ conversion (X_CO2_), selectivity, and yields to products (S_i_ and Y_i_) were determined.^[82]^ They are defined as:

(10) $$X_{{CO}_{2}}=\;\frac{F_{{CO}_{2}in\;-\;}F_{{CO}_{2}out}}{F_{{CO}_{2}in}}$$

(11) $$Y_{i\;}=\;\frac{F_{i}}{F_{{CO}_{2}in\;}};$$

(12) $$S_{i\;}=\;\frac{F_{i}}{F_{{CO}_{2}in\;-\;}F_{{CO}_{2}out}}.$$

### Characterizations Techniques

FT‐IR spectra of fresh catalysts and pure supports were collected with a Nexus Thermo Fisher instrument with 100 scans and spectra resolution of 2 cm^−1^. A pressed disk with less than 0.5 wt % of sample diluted in KBr to a total weight of 1.00 g was used for skeletal spectra analysis.

Online FT‐IR was employed to characterize and assess the behavior of the catalysts during the *in‐situ* drying‐reducing process, carried out for each material before the test. This allowed us to follow the decomposition path of the precursor and possible loss of carbon in the form of either small organic molecules or CO_x_. For *in situ* treatment conditions refer to paragraph 2.3.

X‐ray diffraction patterns of bare support and spent catalysts were obtained using a vertical powder diffractometer, X′Pert. The diffractograms were recorded across the 2θ range of 10–100°, with a scanning step of 0.013° and a time per step that can be varied in the range 1045 ÷ 2550 s, depending on the metal loading of investigated materials. The patterns were indexed by comparing the 2θ values to those documented in Pearson′s Crystal Data database.^[83]^

X‐ray photoemission spectra were recorded using a hemispherical analyzer model 10–360 and a monochromatic X‐ray source model 10–610 by Physical Electronics (AlKα photons, energy hν=1486.6 eV). The instrument is hosted in an ultra‐high vacuum (UHV) chamber with a base pressure better than 5⋅10^−8^ mbar and equipped with a load‐lock system for fast entry of the sample. Photoemitted electrons were collected from a spot of approximately 100 μm diameter. The spectra were analyzed using the KolXPD software, after calibrating the binding energies (E_b_) on the C 1s signal of graphene, set at E_b_=284.5 eV. The C 1s region was fitted by using a Donjac‐Sunich function for the main graphene line, while Voigt functions were employed to fit all the other components. Similarly, Voigt functions were used to reproduce the O 1s, Ni2p, Ni LMM, Ru 3p, and S 2p regions. In particular: i) the Ni 2p spectra were reproduced using three Voigt doublets for metallic Ni and other three for oxidized/hydroxylated Ni^[84]^; ii) the Ru 3d doublet is very close and almost completely covered by the C 1s line. Therefore, only the Ru 3d5/2 component is visible on the low E_b_ side of the C 1s spectrum (E_b_=281.4 eV) and it is fitted with a single Voigt function; iii) the Ru 3p region is fitted with two Voigt doublets.

A Renishaw Raman System 2000 spectrometer with an Ar laser (514.5 nm) excitation source and equipped with a Peltier Cooled CCD as detector and a Leica Optical Microscope was used for carrying out the analysis. Spectra were obtained by focusing the laser through a 50× objective following optimized parameters as an integration time of 20 s, an accumulation number of 4, a Raman shift (from 4000 to 100 cm^−1^), and high gain, keeping off the cosmic ray removal. A filtering system was also applied to vary laser power (100 %, 50 %, 25 %, 10 %, 1 %), but to have comparable data 10 % laser power has been used by making statistical spectra to evaluate the consistency of measures and sample homogeneity.

Microscopic analyses were carried out using an SEM ZEISS SUPRA 40 VP microscope, incorporating a field emission gun (FE‐SEM). This apparatus is equipped with a traditional Everhart‐Thornley and a high sensitivity “InLens” detectors for secondary electrons (SE), a solid‐state detector for backscattered electrons (BSE), and an EDXS (Energy Dispersive X‐ray Spectrometer) OXFORD “INCA Energie 450×3“ for chemical analysis. Both bare supports and exhausted sample powders were sonicated in absolute ethanol; a drop of the obtained suspension was deposited onto a lacey carbon Cu‐grid and then imaged.

Elemental mapping on commercial supports was performed by using a scanning electron microscope (SEM) Zeiss Evo 40 (Carl Zeiss SMT Ltd, Cambridge, England), equipped with an Energy Dispersive X‐ray (EDX) Spectroscope Oxford Instruments INCA X‐ACT. The compositional analysis was carried out based on the characteristic X‐ray intensities of each element, compared with standards (pure elements), and corrected for ZAF effects. A Co standard was used for calibration to monitor the spectrometer′s beam current, gain, and resolution. Specimen powders were directly mounted on a high‐purity conductive double‐sided adhesive carbon tab to be analyzed through SEM‐EDXS.

Moreover, Ni/Com.Gnp and Ni/rGO have been analyzed using a Transmission Electron Microscope (TEM) JEM 2100 Plus supplied by JEOL Ltd (Japan), equipped with a Gatan CMOS Rio9 camera and an EDS Bruker Quantax 200‐STEM microanalysis system. The sample was prepared as done for FE‐SEM observation.

## 2 Results and Discussion

### 2.1 Characterization of the Supports

The obtained supports and starting graphite have been characterized using X‐ray diffraction, Raman Spectroscopy, SEM, FE‐SEM, and XPS.

X‐ray diffraction patterns of C‐based material are reported in Figure S1. Graphite and Gnp show the main features attributable to the presence of the characteristic pattern of graphite and a rhombohedral variant of graphite, with a slight change only in terms of intensity, in line with literature assignments.[^83^, ^85^] It is worth mentioning that no pattern arising from the precursor salt has been observed in line with previous work and adopted preparation procedures. FE‐SEM images of lab‐prepared Gnp are reported in Figure S2, where it can be noticed that a partial exfoliation can be observed, in good agreement with the synthetic route and presented XRD data. Commercial supports i. e., rGO and commercial Gnp display the following quite broad features: 2theta ≈24.8°, 43.2° and 78.5° for the former one and 2theta ≈26.5°, 43.5°, 54.7, and 77.6° for the latter one. These peaks are in line with literature assignations and graphite patterns and can be attributed to the 002, 101, 004, and 110 crystal family planes. Moreover, a broad feature at 2theta~18.5° is also observed, both in Gnp and in rGO patterns, that could be assigned to the presence of disordered carbon as mentioned in the case of fir bark‐derived carbon,^[86]^ being more relevant for commercial C‐derived support, or, as reported, even to the presence of oxygen groups (i. e. hydroxyl‐, epoxy‐hydroxyl, etc.) or to the intercalation of water molecules causing an increase in interlayer spacing up even to 0.53 nm^[87]^ (Figure S1). In Table 1, the 2theta position and the evaluated d_002_ in nm are reported, indicating that the interplanar spacing increases in the order rGO > Com.Gnp≥Gnp≥graphite, in line also with literature.[^87^, ^88^, ^89^, ^90^] The reported data well agree with the ones reported by Chong et al.^[91]^ for commercial graphene nanoplatelets and Gnp‐based materials,^[92]^ suggesting a higher degree of exfoliation when compared with the homemade one. It appears from the discussed data that the used rGO is only partly reduced, showing features assignable to GO, as well.[^93^, ^94^, ^95^] To achieve a clear picture of the starting C‐based catalysts, the elemental composition was investigated by EDX spectroscopy confirming that Gnp materials are quite pure even though a slight presence of S might be envisioned, but in any case, its content is lower than the instrumental detection limit; bare rGO showed instead the presence of Na, Cl, Mn, and S (0.5, 0.1, 0.2 and 0.2 wt % respectively) as main impurities; the mentioned analyses for rGO are included in Figure S3 with the mapping for the mentioned elements.

**Table 1:** XRD data for graphite, as produced Gnp, commercial Gnp, and rGO.

Sample	Notation	2θ of 002 plane (°)	d_002_ (nm)
Commercial Graphite Powder	Graphite	26.60	0.335
Lab‐made Gnp	Gnp	26.56	0.335
Commercial Gnp	Com.Gnp	26.50	0.336
Commercial rGO	rGO	24.86	0.358

**Figure 1:**
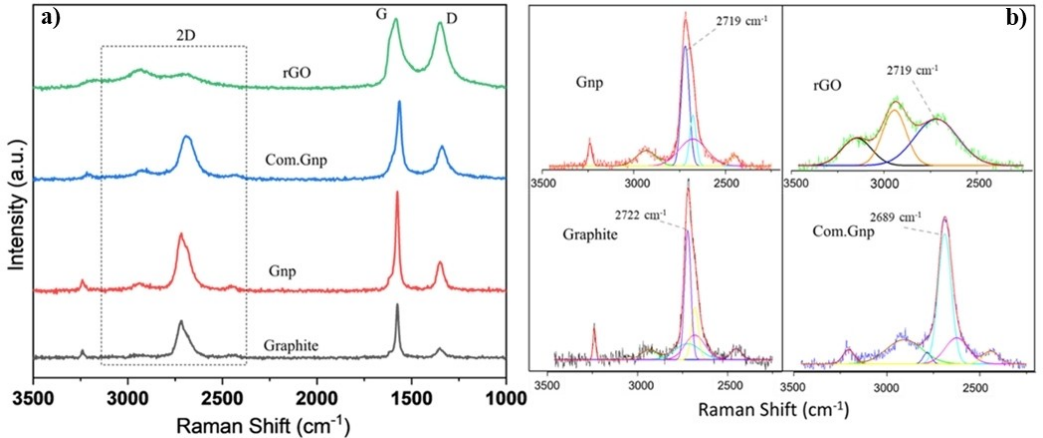
(a) Raman spectra, and (b) deconvolution of Raman spectra of 2D bands of the support materials.

Raman spectroscopy was used to characterize carbon‐based support, focusing on the intensity of the G band, corresponding to sp^2^ carbon bonds in both ring and chain,[^96^, ^97^] and the D peak attributed to the breathing mode of sp^2^ atoms in the ring^[97]^ and to first‐order phonons, indicating in‐plane and edge defects.^[98]^ Moreover, also the 2D peak is of interest considering that it represents the second order of zone‐boundary, composed of two components 2D_1_ and 2D_2_, extremely sensible to the number of layers (up to 10). The ratio of intensities between the D and G bands (I_D_/I_G_) serves as an indicator of defect density[^99^, ^100^] and the Full Width at Half Maximum (FWHM) of Raman spectra is an indicator of the crystalline quality.^[101]^ Furthermore, Raman spectroscopy could be used to calculate both the planar base size (L_a_) of the materials through Equation 13 or Equation 14 (both for L_a_>2 nm)[^93^, ^102^, ^103^, ^104^, ^105^] and their average defect density (n_D_) through Equation 15 and average distance between defects (L_D_) (Equation 16[^106^, ^107^]

(13) $$L_{a\;}\left(nm\right)=\;\frac{2.4\;\times 10^{-10}\;{\lambda_{laser}}^{4}}{\left(I_{D}/I_{G}\right)}$$

(14) $$L_{a\;}\left(nm\right)=\;\frac{560}{E_{\lambda }^{4}\left(I_{D}/I_{G}\right)}$$

(15) $$n_{D\;}\left({cm}^{-2}\right)=\;\frac{\left(1.85\;\pm 0.5\right)\;\times 10^{22}}{{\lambda_{laser}}^{4}}\left(\frac{I_{D}}{I_{G}}\right)$$

(16)

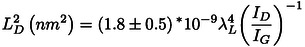




where λ_laser_ is the wavelength of the Raman laser source and E_λ_ is the excitation energy of the laser in eV (2.41 eV in this work). Raman spectra of support material are shown in Figure 1, after careful baseline subtraction. In all cases, the main characteristic bands are found even though with changes in the observed position. In the case of commercial graphite, features at 3238, 2717, 1577 and 1349 cm^−1^ have been observed together with broad shoulders at 2942 cm^−1^ and 2449 cm^−1^, in line with the classical 2D (2710 cm^−1^), G (1580 cm^−1^) and D (1330 cm^−1^) features, together with the appearance of the second order D’ intravalley peak noted as 2D’(3250 cm^−1^)[^88^, ^108^, ^109^, ^110^, ^111^] and of D+D’ feature (2940 cm^−1^).^[106]^ In addition, for commercial graphite a broad shoulder at 1618 cm^−1^ also appears and is assigned to the presence of defective graphite, for the presence of phonons with a small q (wavevector). As it has been reported,^[112]^ the 2D band in bulk graphite originates from the convolution of an infinite number of peaks. Hence, the deconvoluted 2D band is also shown in Figure 1 to present a comprehensive representation of this peak in various supports; in the case of graphite, the I_D_/I_G_ ratio is 0.2 and I_2D_/I_G_ is in the range of 0.5–0.7 suggesting slight heterogeneity of the sample, in line with the multilayer nature of the investigated material. Home‐made Gnp shows features at 3238, 2712–2719, 2445, 1569–1575 and 1344–1350 cm^−1^. An evaluation of multi‐point analysis allowed us to obtain an I_D_/I_G_ =0.30 with only a few analyses where this ratio is reduced to 0.08–0.10, in line with the multilayer nature and with the slight heterogeneity of the produced material.

On the other hand, Com.Gnp exhibits more structural disorders than Gnp, while, as expected, rGO demonstrates the most defective structure. Com.Gnp showed a different position for the 2D band that appears now shifted to 2696–2681 cm^−1^, in line with the conventionally observed shift for fewer layers^[97]^; moreover, the G and D bands are found at 1576–1565 cm^−1^ and 1338–1342 cm^−1^, respectively. The observed I_D_/I_G_ ratio is equal to 0.41–0.75 while the I_2D_/I_G_=0.58–0.63, in line with previously discussed data and accounting for the fact that the profile of the 2D mode in graphene is strongly dependent on the number of layers.^[113]^

**Figure 2:**
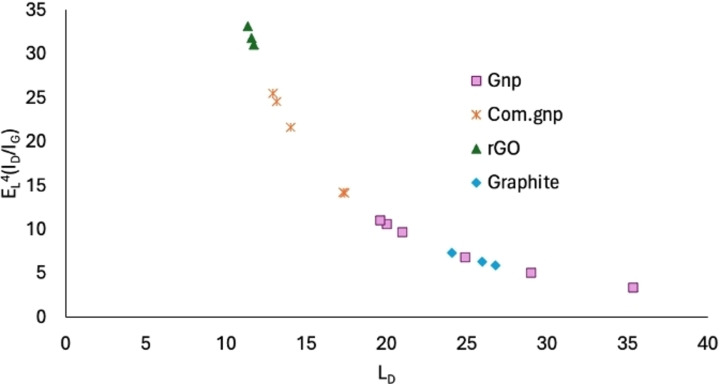
Number of defects as a function of the distance of defect L_D_ [nm] for the investigated materials.

rGO displays a random relative stacking of the graphene layers, thus causing an increase in the peak widths of all modes with the disorder. Main bands are found at 3149–3177, 2927–2940, 2693–2704, 1584–1582, and 1350 cm^−1^, with again a broad shoulder at 1618–1620 cm^−1^. The first three features lead to an overlap of the 2D mode with other modes (D+D′ and 2D′), resulting in a broad bump‐like feature in the ∼2500–3200 cm^−1^ range,^[114]^ even though the peak at 2950 cm^−1^ is assigned to both the defective activated D+D’ and D+G mode.^[115]^ The observed I_D_/I_G_ ratio is equal to 0.92–0.98 while the I_2D_/I_G_=0.19–0.25, in line with literature results and with a possible system displaying a partial reduction,^[116]^ accounting for the fact that for crystallite sizes lower than 2 nm an opposite trend is frequently observed for I_D_/I_G_, i. e., decreasing with an increasing disorder.^[117]^ The calculated crystallite size and defect density of rGO are in line with those reported in the literature.^[88]^ Moreover, for a better understanding, the L_D_ has been calculated as proposed in reference^[106]^ (Equation (16)) and the results are reported in Figure 2, where it is possible to outline an increased number of defects (reduction of L_D_) in the order graphite<Gnp<Com.Gnp<rGO and mostly following the shaded region proposed for E_L_^4^(I_D_/I_G_) vs. L_D,_ accounting for the experimental error.^[106]^ Moreover, the presence of other atoms could induce a shift in the position of the observed bands with the appearance of C−S interaction at 1530 cm^−1^, in agreement with ν_3_ vibrational modes observed in CS_2,_ ^[118]^ thus allowing to mention this possible contribution in the described spectra for Gnp and rGO materials; it is worth noting that the position for G and D bands in rGO well plays with S‐containing ones.[^119^, ^120^] Data are also summarized in Table 2.

**Table 2:** Raman data for graphite, Gnp, commercial Gnp (Com.Gnp) and rGO. Peak positions have been obtained on the unsubtracted spectra and acquired data points.

				Raman Shift (cm^−1^)		
Name	I_D_/I_G_	n_D_ (cm^−2^) (10^11^)	L_a_ (nm)	2D	D	G
Gnp	0.10–0.33	0.27–0.87	53–166	2713–2720	1344–1349	1569–1576
Com.Gnp	0.42–0.76	1.11–2.01	22–40	2681–2696	1338–1342	1565–1576
rGO	0.92–0.99	2.44–2.61	17–18	2694–2704	1351	1582–1584
Graphite	0.18–0.22	0.47–0.58	75–89	2717	1348	1576

FT‐IR spectra of investigated materials are reported in Figure S4. Graphite and homemade produced Gnp displayed only weak features at 1620, and 1590 cm^−1^ together with a broad at 1390 cm^−1^, in line with classical features associated with δ_OH_ (of adsorbed water in KBr), asymmetric and symmetric vibration modes of groups (C−H) and −C=C− of aromatics. The broad components at 1060 cm^−1^ and 1380 cm^−1^ are assigned to C−O and C−OH vibration, respectively. In the Com.Gnp case, features in 1710, 1635, 1575, and 1222 cm^−1^ are present, also suggesting the presence of the C=O group (1710 cm^−1^), as indeed expected. However, a more complex spectrum is observed for rGO, in line with XRD and Raman data, where bands at 1720 and 1590 cm^−1^ together with a broad and multicomponent feature centered at 1150 cm^−1^ are observed, in line with the results reported in the literature for GO and rGO with the presence of C=O, O−C=O and C−O−C bonds.^[121]^

**Figure 3:**
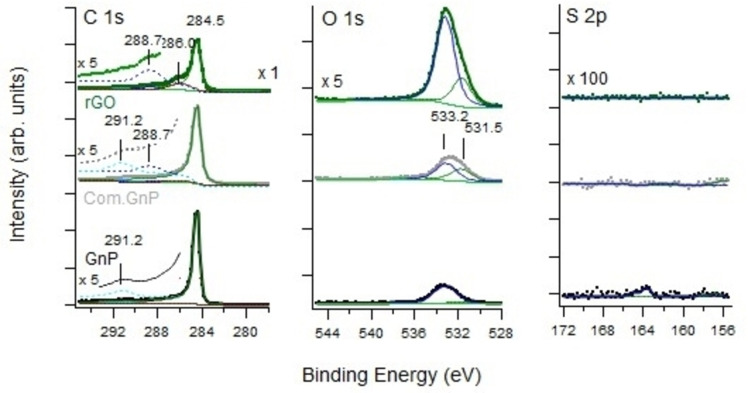
XP spectra in the region of C1s, O1s, and S2p for the investigated supports. We underline that the O 1s intensity scale is multiplied by a factor x5, the S 2p is multiplied by a factor x100 with respect to the C 1s traces for sake of clarity. The high E_b_ tail of the C 1s region is also multiplied by a factor x5 with respect to the corresponding C 1s trace.

To further investigate the starting supports, XPS analyses on the investigated materials have been carried out. In Figure S5, the survey spectra are included, while C1s, O 1s, and S 2p are shown in Figure 3.

The C1s region is characterized by a main line at 284.5 eV, corresponding to sp^2^ carbon and thus assigned to graphene/graphite nanoplatelets. The peaks at higher E_b_ are related to the presence of C−O, C=O, O−C=O, and C−OH bonds and are particularly evident for rGO, suggesting only a partial reduction in line with Raman data, literature,[^117^, ^122^, ^123^, ^124^, ^125^] and FT‐IR spectra. The nearly vanishing intensity around 291 eV could tentatively be assigned to carbonates, even though not clearly detected by other techniques. The feature at 286 eV is compatible with C−OH and C−O−C group and is typically present in GO,^[122]^ as from the hypothesis of partially reduced materials supported by other characterization techniques. The O 1s region of the pure supports is characterized by two main contributions at 531.8 and 533 eV. The O 1s region of the pure supports is characterized by two main contributions around 531.5 eV and 533.2 eV. The intensity of the former line is higher for rGO and for the Com.Gnp, in accord with the larger intensity of the high E_b_ components observed in the corresponding C 1s spectra and with the complex feature in the 2400–3000 cm^−1^ region in vibrational spectra.

Its E_b_ is close to the one (532.0) reported by Mohai et al.,^[122]^ who assigned it to O in ether or epoxy, OH in alcohol, C=O in ester. Its presence is correlated with the appearance of the peak at 288.7 eV (assigned to carboxyl or ester in^[122]^). The same E_b_ might also be compatible with oxygen in carbonates^[126]^ but this assignment is less likely since the peak 291 eV is evident also for Gnp, where the contribution at 531.5 is definitely lower.

The 533.1 eV component, the largest for rGO, is assigned to water, even though in some cases assignation to the carbon‐oxygen functional group has been reported.[^64^, ^77^, ^82^, ^127^] The S 2p region shows a weak intensity around 164 eV only for the Gnp sample. It is compatible with slight S doping of the C‐based material, in accord with the value of 163.9 eV reported for the S 2p2/3 line of S‐doped G.^[128]^

### 2.2 Catalysts Characterization and Performance in CO_2_ Hydrogenation

**Figure 4:**
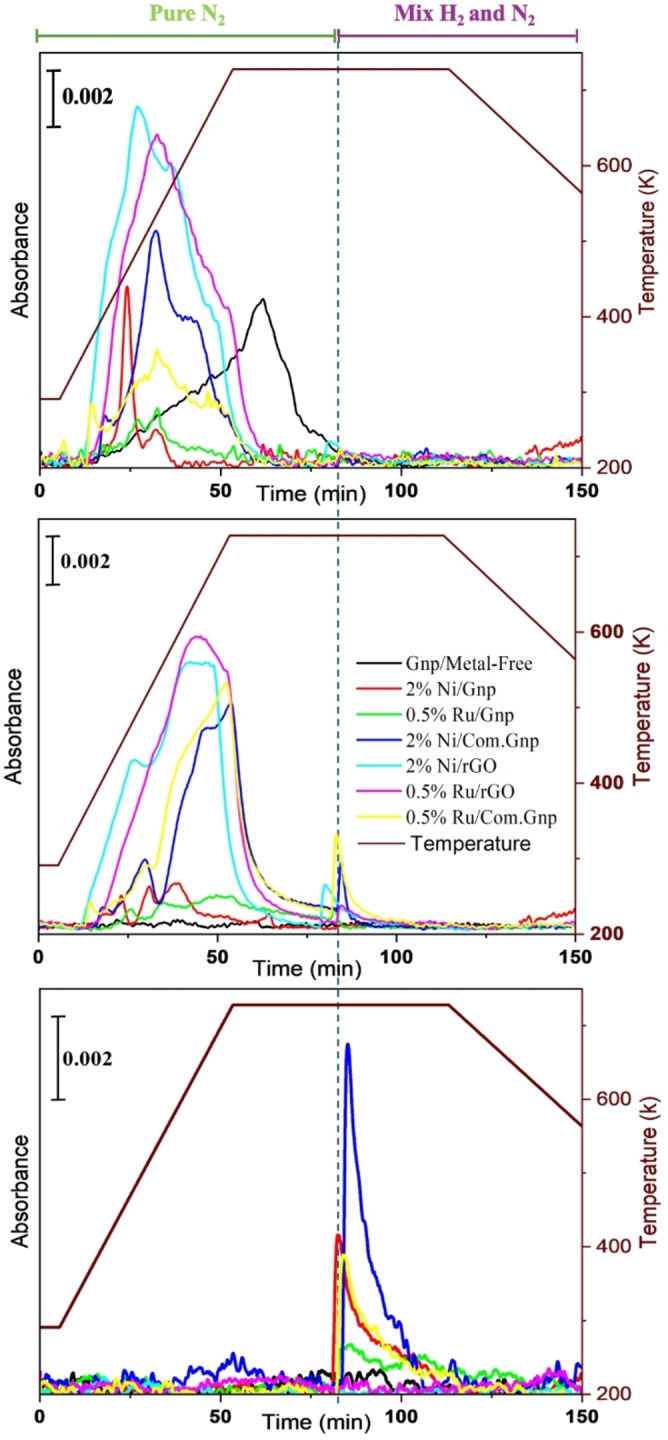
Absorbance profiles of (a) CO_2_, (b) CO, and (c) CH_4_ as a function of time at 2293, 2170, and 1333 cm^−1^, respectively. The corresponding temperature profile is also included.

The recording of *in situ* pre‐reduction aimed at investigating various aspects of the catalyst, including support stability, NO_x_ release and evolution (originating from metal precursors nitrate salts), and potential side reactions occurring during reduction, such as hydrogenation, partial oxidation etc. The stability of carbon materials is a concern when dealing with thermal CO_2_ conversion reactions. In assessing the stability of the carbon‐based catalyst during reduction (the initial thermal treatment of the synthesized catalyst), the potential production of carbon‐containing products such as CO_2_, CO, and CH_4_ was examined. Therefore, the FT‐IR profiles over time for these specified molecules were analyzed and presented in Figure 4 and quantitative determinations have been carried out on CO_2_, CO, and CH_4_ generated during the reduction process, thereby calculating the effective C‐loss as reported in Table 3.

**Table 3:** Calculated carbon loss of each catalyst by producing CO_2_, CO, and CH_4_ during the reduction process.

Catalyst	Produced Gas	Amount produced (mole)	Carbon loss (mole %)	Total Carbon Loss (mol. %)	Total Carbon Loss (mg)
Gnp/Metal‐Free	CO_2_	3.25 E−05	0.4 %	0.4 %	0.4
	CO	0.00 E+00	0.0 %		
	CH_4_	0.00 E+00	0.0 %		
2 % Ni/Gnp	CO_2_	2.32 E−05	0.3 %	0.7 %	0.5
	CO	7.65 E−06	0.1 %		
	CH_4_	1.28 E−05	0.2 %		
2 %Ni/Com.Gnp	CO_2_	9.69 E−05	1.3 %	3.1 %	2.7
	CO	7.80 E−05	1.1 %		
	CH_4_	5.27 E−05	0.7 %		
2 %Ni/rGO	CO_2_	2.20 E−04	3.0 %	4.8 %	4.2
	CO	1.24 E−04	1.7 %		
	CH_4_	6.64 E−06	0.1 %		
0.5 %Ru/Gnp	CO_2_	9.21 E−06	0.1 %	0.5 %	0.4
	CO	1.54 E−05	0.2 %		
	CH_4_	1.33 E−05	0.2 %		
0.5 %Ru/Com.Gnp	CO_2_	6.66 E−05	0.9 %	2.7 %	2.4
	CO	9.34 E−05	1.3 %		
	CH_4_	3.75 E−05	0.5 %		
0.5 %Ru/rGO	CO_2_	1.98 E−04	2.7 %	4.4 %	3.
	CO	1.24 E−04	1.7 %		
	CH_4_	0.00 E+00	0.0 %		

To the best of our knowledge, this study represents the first investigation into the stability of novel carbon‐based catalysts used for CO_2_ hydrogenation in real reduction conditions and the findings offer valuable insights. It was observed that the carbon loss in Gnp support was lower compared to Com.Gnp and rGO. This observation aligns with the Raman results (Figure 1), which indicated that in terms of defects, the order was rGO>Com.Gnp>Gnp. Additionally, it was evident that the rGO‐based group tended to lose carbon primarily through the release of CO_x_. This aligns with their defective plane condition, which is characterized by the presence of oxygen‐containing functional groups such as carboxyl groups.[^129^, ^130^] Note that the potential occurrence of the reverse Boudouard reaction[^131^, ^132^] under the test conditions was not taken into account in this methodology for the remarkable CO_2_ partial pressure.

**Figure 5:**
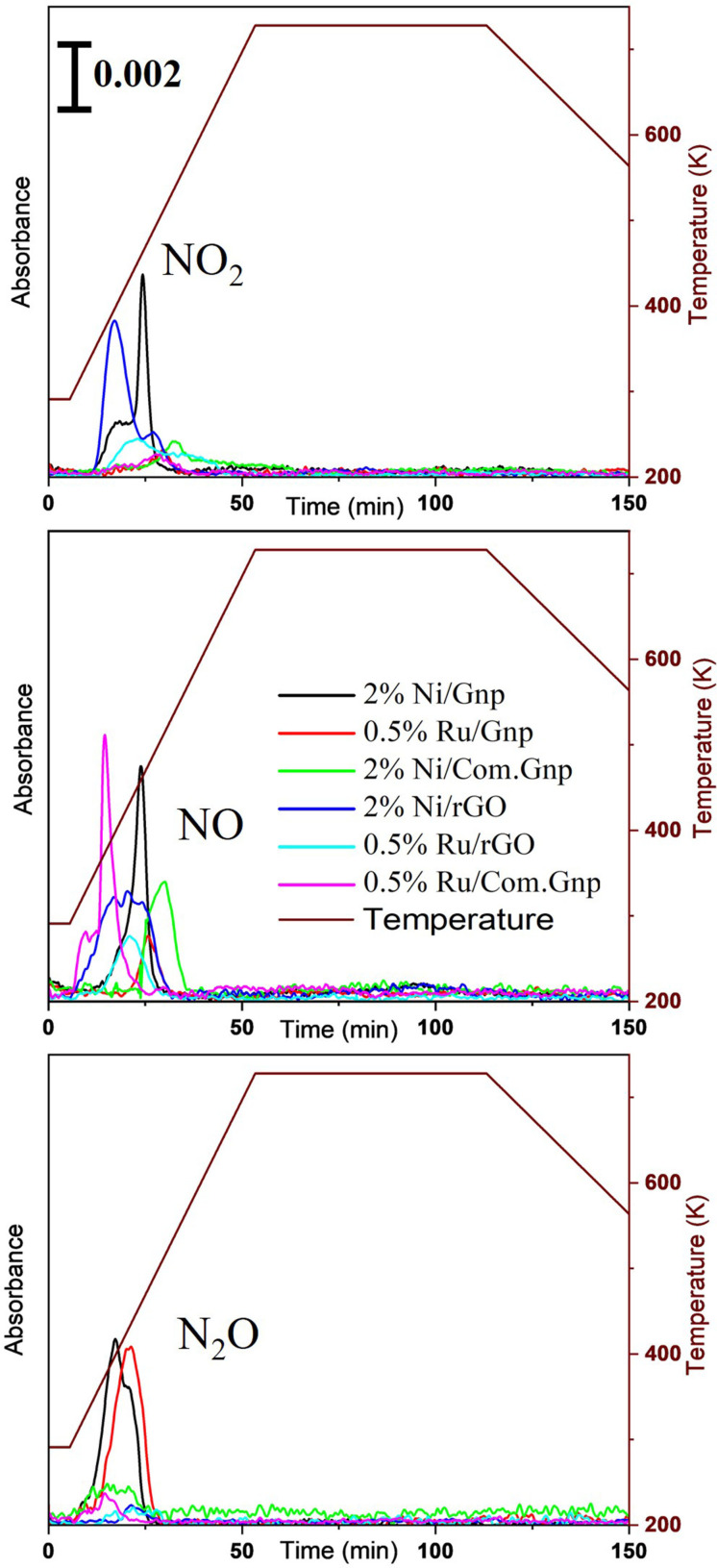
Absorbance profile of NO_x_ released during the reduction of catalysts and the corresponding time and temperature. Profiles of (a) NO_2_ at 1587 cm^−1^, (b) N_2_O at 2239 cm^−1^, (c) NO at 1890 cm^−1^.

The analyzed results of NO_x_ release from the as‐prepared catalysts during the reduction process are depicted in Figure 5.

The generation of NO_x_ in this context originates from the metal precursor nitrate salts and, in line with literature data, NO is produced from Ni(NO_3_)_2_*6H_2_O at 420 K^[133]^ according to the reaction:

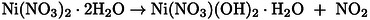




that, in the presence of water, can give rise to side reactions such as:

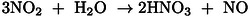




and accounting also for 3NO→NO_2_+N_2_O.

According to literature data, the full decomposition of the starting Ni‐precursor, in the N_2_ atmosphere, mainly produces NiO above 523 K, while it gives metal particles in the case of H_2_/N_2_ mixtures,^[133]^ thus helping in reducing C‐loss. In the Ru‐case, a quite relevant NO_x_ production is observed at ~433 K in line with the decomposition of ruthenium nitrosyl nitrate with NO production, as from TGA data^[134]^ for RuO_x_ nanoparticle synthesis. The overlapping of NO_x_ and CO_x_ profiles allows to hypothesize the occurrence of NO_x_ reaction at the expense of CO produced, according to the following reactions catalyzed by Ni and Ru^[135]^:

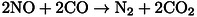




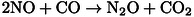




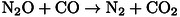




where N_2_O concentration is negligible above 453 K in agreement with Forzatti′s work on Pt−Ba/Al_2_O_3_ as LNT (lean NO_x_ traps) catalysts, and where N_2_O was also reported to be able to oxidize Pt to PtO.^[136]^ Bare Gnp (Gnp/Metal‐Free) does not display the NO_x_ feature and only CO_2_ is observed, with a maximum concentration in the dwelling stage i. e., at higher temperatures.

Also, despite the presence of Ru and Ni upon introduction of H_2_ at the maximum temperature, no ammonia is evolved suggesting the full decomposition of nitrate and a possible surface reorganization of S upon treatment, considering that online FT‐IR has outlined no S‐containing compound, in remarkable amounts, in the gas phase. This would in principle support a slight decrease in hydrogenation activity also in line with the observed behavior for CO and CH_4_ profiles, as indeed observed for Co‐based materials.[^137^, ^138^, ^139^, ^140^, ^141^, ^142^, ^143^] Upon dwelling, the production of CO and CH_4_ is reduced to zero in the case of rGO materials, suggesting a quite different catalytic system.

#### 2.2.1 Catalytic Results in CO_2_ Hydrogenation

**Figure 6:**
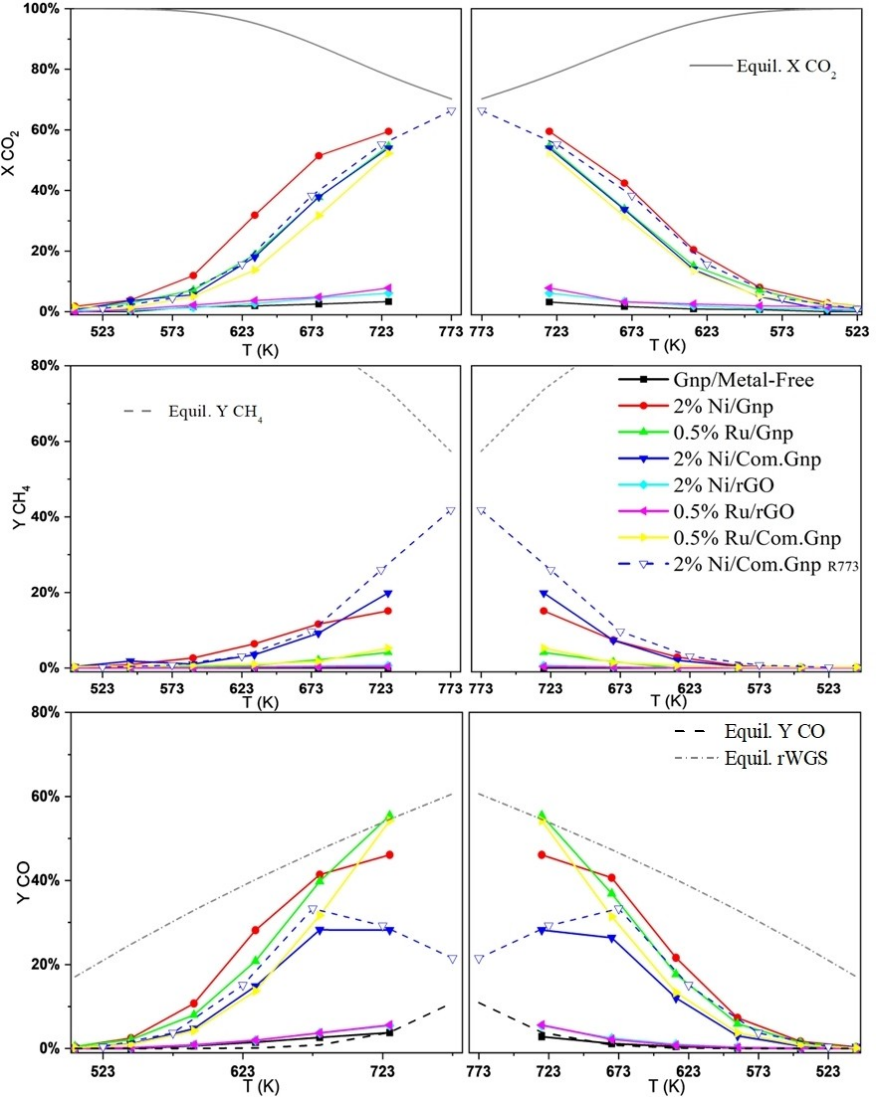
CO_2_ conversion, CH_4_ and CO yields in CO_2_ hydrogenation on investigated materials and the forecasted thermodynamic equilibria (METH+rWGS ‐ Equil. X CO_2_, Y CH_4_, Y CO) and (rWGS ‐ Equil. rWGS) in the temperature range of 523–773 K.

The catalytic activity of the prepared catalysts in CO_2_ hydrogenation is shown in Figure 6, in terms of CO_2_ conversion, CO, and CH_4_ yields.

The findings indicate that Gnp/Metal‐Free exhibited almost no activity (maximum CO_2_ conversion 5 % to CO), along with the catalysts prepared with rGO i. e., 2 % Ni/rGO, and 0.5 % Ru/rGO that were inactive in Sabatier′s reaction displaying only weak activity in reverse Water Gas Shift (rWGS) with a maximum yield to CO of 7 % at 723 K, irrespective of using Ni or Ru as active phases. All catalysts resulted mostly inactive in the temperature range 500 K–570 K, while at higher temperatures performances remarkably differed. Comparing Ni‐based catalysts supported on Gnp and Com.Gnp, it was observed that 2 % Ni/Gnp exhibited higher activity in CO_2_ conversion compared to 2 % Ni/Com.Gnp with a 10 % CO_2_ conversion at 590 K and increasing with temperature up to 60 % at 723 K with CO as the main product, being CH_4_ a by‐product with maximum yield (17 %) at 723 K. However, it is interesting to remark that slight deactivation occurs on each of the steps in terms of CO_2_ conversion, producing reduced performances in the decreasing temperature experiments that well mimic the performance obtained for 2 % Ni/Com.Gnp both in the same testing conditions and isothermal ones. Ni/Com.Gnp has been tested in isothermal and on gradient temperature reactors and the main difference is observed at high temperatures, where slightly higher performances in terms of conversion and yield are observed mainly toward methane at high temperatures and CO at lower ones (XCO_2_=66 %, YCH_4_=42 %, and YCO=21 % at 773 K). These materials resulted more active, in terms of conversion and CO yield, than Ni/SiO_2_ and Ni/SiO_2_‐Al_2_O_3_ catalysts tested in analogous experimental conditions and similar Ni loading.^[50]^ According to^[144]^, for Ni/SiO_2_ a critical diameter for the switch in‐between rWGS and methanation is present and set at 9 nm, evidencing that low loading Ni‐catalysts are more selective toward CO, suggesting different pathway are present even though the support and preparation procedure might play a role.^[145]^ As well reported data well play with results obtained for single atom in N‐doped carbon nanotube.^[146]^

Ru‐based catalysts supported on Gnp and Com.Gnp displayed interesting CO_2_ conversion within the tested temperature range (500–725 K). In particular, for Ru/Gnp in the increasing temperature experiment conversion in zero below 523 K while it starts to increase achieving its maximum at 723 K with the production of CO as the main product; methane is only observed as a side product above 700 K with a maximum yield of 5 %. A similar behavior is also obtained for Ru/Com.Gnp catalysts even though in this case a slightly reduced conversion is obtained but with a similar distribution in term of products. In the decreasing temperature experiment, the performances are kept constant, even though it is possible to envisage that the conversion and yield profiles for CO_2_, CO, CH_4_ are overlapped for the two investigated materials. Thus, below 670 K, both Ru‐based catalysts are completely selective in CO production (100 % selectivity) while above also methanation is occurring, even though with a low extent of reaction. Moreover, both catalysts approach the forecasted thermodynamic equilibrium at the maximum temperature, ranking thus high the activity of these materials. However, Ru− is a well‐known active phase for CO_2_ hydrogenation to methane if Ru loading is 0.5–5 wt %[^147^, ^148^, ^149^] but in the present case CO is observed as main product. Similar activity has been reported for overcoated Ru/SiO_2_ catalysts (H‐SiO_2_@Ru@SiO_2_) with a possible role of the confinement and of narrow particle size distribution for Ru− in hollow silica^[150]^ or with the application of an electric field for Ru/ZrTiO_4_ assigned to the virtue of promoted hydrogen migration (surface protonics) on Ru surface.^[151]^ As indeed mentioned for support, the slight presence of sulphur could also contribute into the observed catalytic activity for both Ru− and Ni− catalysts, thus investigated in the exhaust material. Due to the novelty of the proposed catalytic system, apparent activation energy for rWGS has been also evaluated and the following values have been obtained from data refinement: 91 kJ/mol for Ni over both Com.Gnp and Gnp, 75 kJ/mol, and 88 kJ/mol for Ru/Gnp and Ru/Com.Gnp while a quite lowered one has been obtained for rGO based system i. e., 58 kJ/mol for both Ru− and Ni− catalysts. In the case of Ni over graphene nanoplatelets catalysts, the activation energies are in line with those reported for Ni/Al_2_O_3_ catalysts[^23^, ^148^] and sulfided Ni/Al_2_O_3_, in line with literature observations.^[152]^

### 2.3 Characterization of Exhaust Catalytic Materials

**Figure 7:**
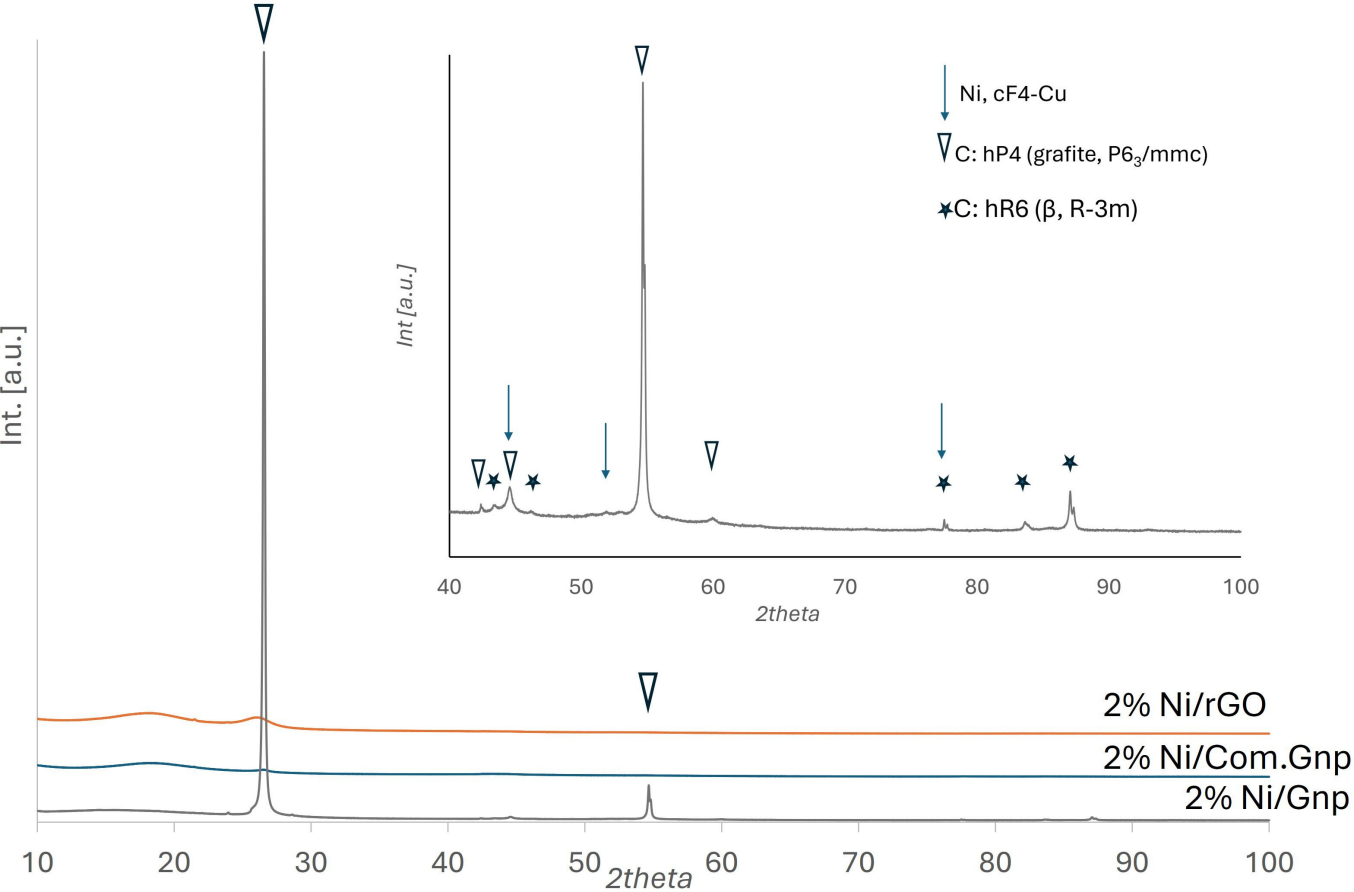
XRD patterns of Ni‐based catalysts. In the inset, the 2% Ni/Gnp pattern in the 2theta range 40–100 is shown with an enlarged intensity scale.

**Figure 8:**
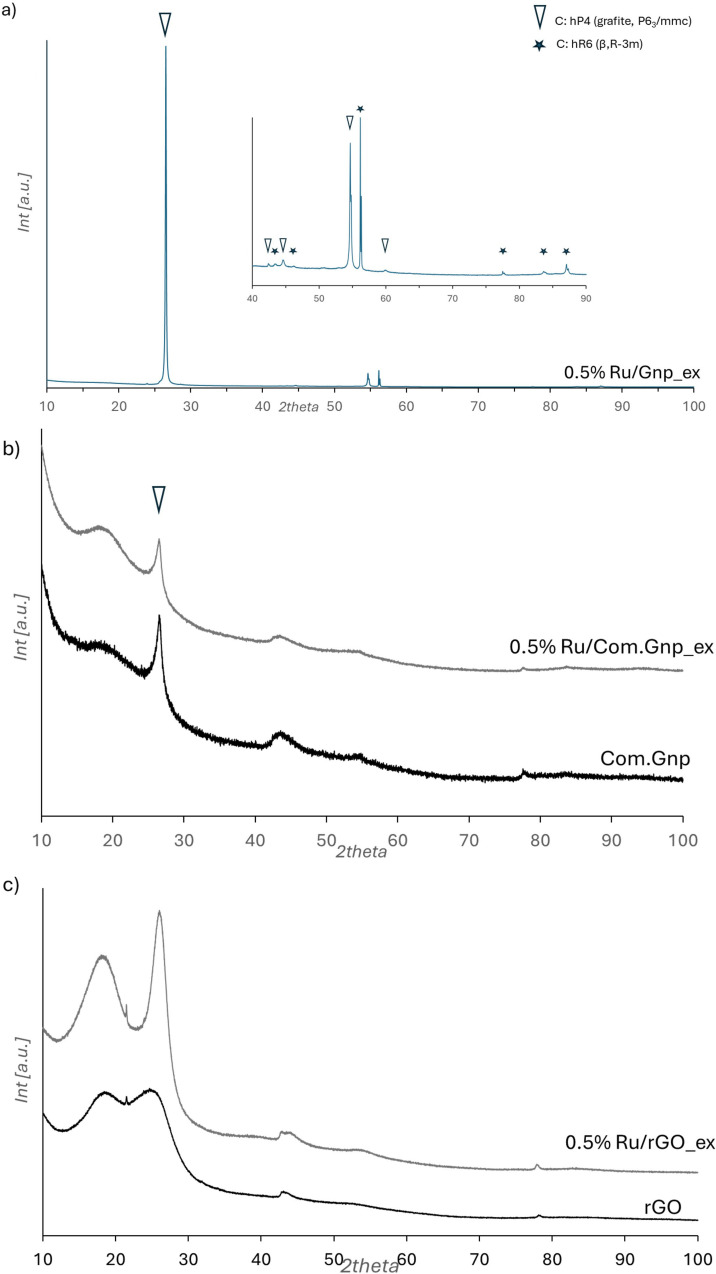
XRD patterns of Ru‐based catalysts: (a) 0.5% Ru/Gnp pattern ‐ in the inset, the pattern is shown with an enlarged intensity scale; (b) 0.5% Ru/ Com.GnpG ex and its bare support; (c) 0.5% Ru/rGO ex and the corresponding support.

X‐Ray diffraction patterns of exhaust catalytic materials are reported in Figures 7 and 8 for Ni and Ru, respectively.

Ni‐ and Ru‐Gnp materials showed the classical diffraction pattern of graphite, to which is superimposed the β‐pseudo graphite variant pattern whose features are the following, with, in parenthesis, the crystal family planes: 26.56° (003), 43.36 (101), 46.22 (012), 56.56 (104) 77.52 (110), 83.64 (113), 87.12 (009).^[83]^ This β form is rhombohedral (space group *R* $\bar{3}$ *m*), and its unit cell has a c‐axis 1.5 times as long as the ordinary graphite. In the XRD patterns of the other catalysts supported on Com.Gnp and rGO, still, two contributions are found in the 2theta region 15–27°, with a more defined diffraction pattern at 2theta≈18° and the quite remarkable definition of a sharp peak at 2theta≈26.1÷26.5° for rGO and Com.Gnp, respectively, that can be attributed to graphite, suggesting a partial loss of the original functional group and a partial increase of stacking, especially for the catalysts supported over rGO. This fact is also supported by the significant increase in interplanar spacing for catalysts supported on rGO, as shown in Table S1, where the related 2theta position and the evaluated d_002_ in nm are reported. Moreover, no features assignable to Ru‐containing phases is observed, while in the case of Ni‐based samples, the most intense peak related to metallic Ni could be envisaged in the region centered at 2theta=44.5°, even though the assignation is doubtful for the overlapping with the 101 of graphitic crystalline phases.

**Figure 9:**
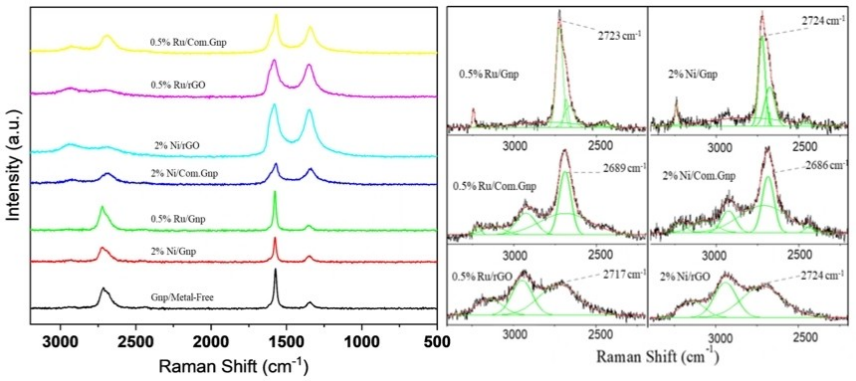
(a) Raman spectra, (b) magnified view of deconvoluted data of Raman spectra at 2D band region.

The Raman spectra of exhaust catalytic materials are reported in Figure 9 and relevant data are summarized in Table 4.

**Table 4:** Raman data the spent (after catalytic activity) catalysts. Peak data have been reported for the unsubtracted spectra and by accounting for all collected data points.

				Raman Shift (cm^−1^)		
Sample	I_D_/I_G_	L_a_ (nm)	n_D_ (cm^−2^) (10^11^)	2D	D	G
Gnp Metal‐Free	0.16–0.40	41–104	0.54–1.06	2713–2720	1344–1351	1573
2 % Ni/ Gnp	0.22–0.33	51–76	0.58–0.87	2719–2722	1351–1355	1576–1578
0.5 % Ru/ Gnp	0.12–0.18	94–138	0.30–0.47	2717–2724	1353–1355	1576
2 %Ni/Com.Gnp	0.62–0.76	22–27	1.52–2.01	2687–2694	1338–1342	1569–1571
2 %Ni/rGO	0.87–0.92	18–19	2.13–2.43	2683–2691	1346–1353	1582
0.5 % Ru/ rGO	0.88–1.00	17–19	2.13–2.66	2681–2707	1344–1351	1582–1584
0.5 %Ru/Com.Gnp	0.44–0.86	19–38	1.08–2.27	2683–2700	1338–1344	1567–1572

**Figure 10:**
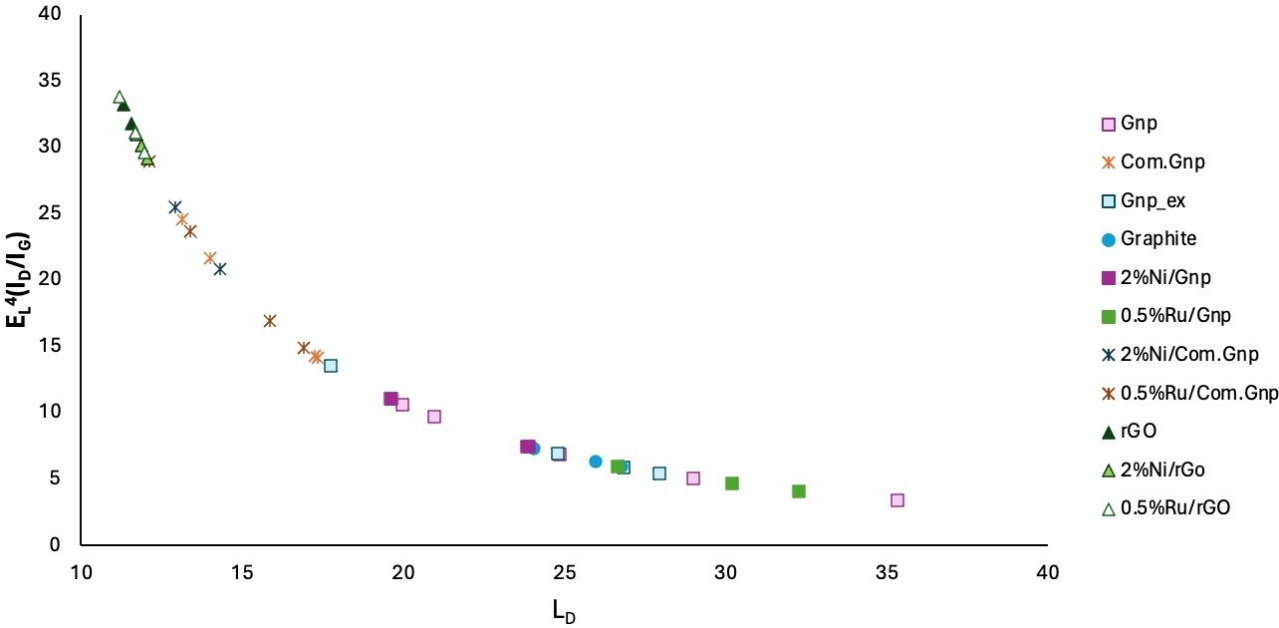
Number of defects as a function of the distance of defect L_D_ [nm] for the investigated catalytic materials after CO_2_ hydrogenation reaction.

In all cases, the exhaust materials show quite similar spectra when compared to the corresponding original used support, suggesting the right choice for a reliable comparison when accounting also for C‐loss in reducing environment; however, some slight changes in the position of D and G bands might be observed together with slight changes in the I_D_/I_G_ ratio. Moreover, for all samples, the band at 1605–1620 cm^−1^ appears more evident in line with the original assignation of defective graphite and discussed XRD data and IR spectra of exhaust materials (Figure S4) where the bands assigned to C=O completely disappeared with the presence of some other broad and weak ones. In line, the plot of the density of defects and their distance is reported in Figure 10. It is possible to observe that the obtained data fit well in the forecasted curve. The introduced points still rely on the same ones and in the characteristic range of the starting support, even though, for Ru and Ni‐Gnp materials, a higher variation of L_D_ is observed in line with the heterogeneity that characterized this system. In contrast, in the case of rGO as a support, Ru reduced L_D_ while on nickel the distribution is apparently shifted to higher ones. The greatest variability has been indeed observed for Ni/Gnp material in line also with the more pronounced deactivation and heterogeneity.

**Figure 11:**
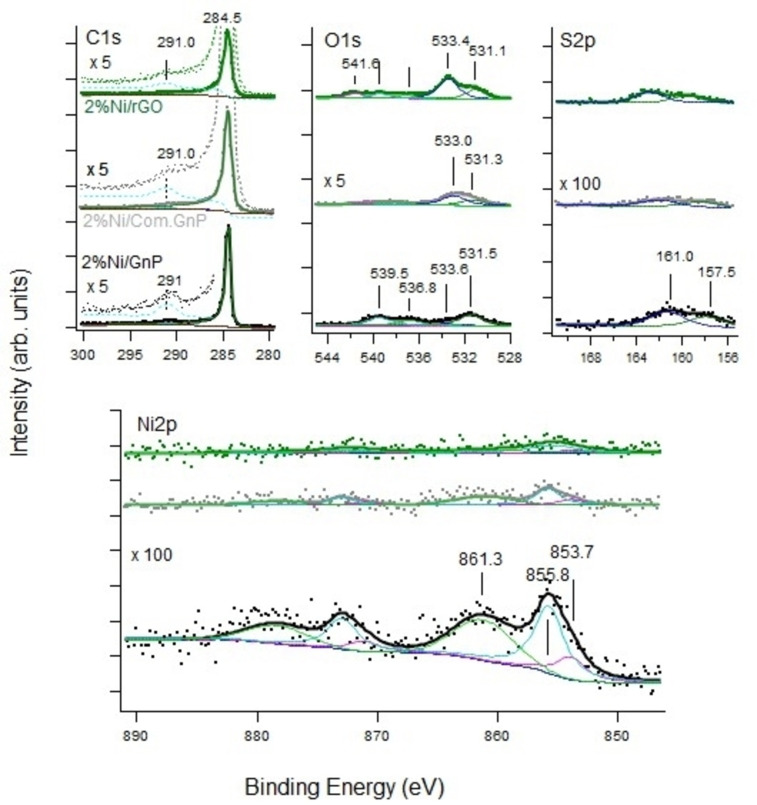
Upper panels: XPS spectra of the C 1s (left), O 1s (center), and S 2p (right) regions and Lower panel: panel: Ni 2p spectra recorded for the different samples. We underline that the O 1s intensity scale is multiplied by a factor ×5, the S 2p and Ni 2p intensities are multiplied by a factor ×100 with respect to the C 1s traces for sake of clarity. The high E_b_ tail of the C 1s region is also multiplied by a factor x5 with respect to the corresponding C 1s trace.

**Figure 12:**
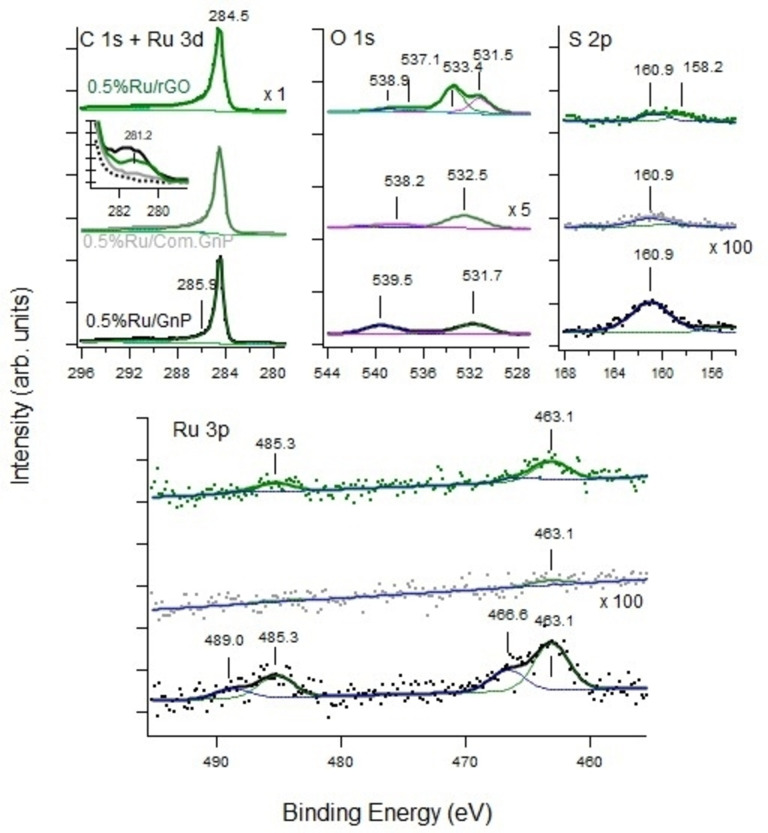
Upper panels: XPS spectra of the C 1s (left), O 1s (center), and S 2p (right) regions and Lower panel: Ru 3p spectra recorded for the different samples. The inset shows the Ru 3 d component not overlapped by the C 1s line. We underline that the O 1s intensity scale is multiplied by a factor ×5, the S 2p and Ru 3p intensities are multiplied by a factor ×100 with respect to the C 1s traces for sake of clarity.

The XP spectra of interest for both Ni‐ and Ru‐based catalysts are reported in Figures 11 and 12, respectively. The corresponding surveys are reported in Figures S6 and S7.

For the Ni‐loaded samples, the C 1s region is dominated by the main line at 284.5 eV, corresponding to sp^2^ carbon in graphene/graphite nanoplatelets; apparently, the spectra do not change with respect to the corresponding bare supports (see Figure 3), suggesting possibly a weak interaction of the nanoparticles with them. The O 1s region should be compared with the one previously described for bare support and it is possible to notice that the exhausted Ni‐loaded catalysts present additional features at 531.1, 536.8, 539.5, and 541.6 eV, the nature of which will be discussed in the following. The Ni 2p signal is relevant only for the Ni/Gnp sample, while its intensity is much lower for the other two samples, despite the same nominal amount of Ni nanoparticles. Similarly, only the Auger Ni LMM transitions of the Ni/Gnp sample are detectable (Figure S8 for Ni LMM). Considering that the orientation of the Gnp is random and that XPS analysis is sensitive to the topmost surface layers, this observation suggests that Ni nanoparticles populate more densely the borders of the nanoplatelets in the Gnp support. The fitting of the Ni 2p region indicates that Ni is oxidized or hydroxylated, since the fingerprint of metallic Ni 2p (Ni 2p_3/2_ lines around 852.7 eV, 858.7 eV) is indeed missing. By comparing the Ni 2p lines with those reported for NiO, Ni(OH)_2,_ and γ‐NiOOH,[^84^, ^128^] it is evident that our spectra are compatible with all three species since all of them present a maximum of intensity around 855.5 eV. However, the corresponding O 1s spectra present a significant component at 531.1 eV that is compatible with Ni hydroxylation, while the signal expected around Eb=529.2 eV for NiO is missing. Thus, Ni nanoparticles might be in the form of Ni(OH)_2_ and/or γ‐NiOOH, but NiO is not present in a significant amount, in line with all other techniques. To further clarify this point, we report in Figure S8 the Ni LMM auger spectra for the different samples. Coherently with the Ni 2p XPS data, only the Ni/Gnp sample presents a significant intensity of the Auger peaks at kinetic energy E_kin_=708, 772, and 843 eV. This last value is in good agreement with the one reported by Biesinger et al. for Ni(OH)_2_ (842.58 eV).^[128]^ Comparison with the Wagner plot for Ni^[128]^ confirms this identification. The surveys of Figure S6 allow us to investigate the presence of other minor contaminants; indeed, they show traces of N (N 1s line at 400 eV) and S (S 2p lines at ~160 eV), while the presence of other common contaminants as K and Na is ruled out.

Since it is well established that even a small sulfur concentration can severely affect the reactivity of Ni, high‐resolution spectra of the S 2p region have been recorded for the exhausted Ni‐loaded samples. Different S components show up with S 2p_3/2_ lines at 161.0 eV and 157.5 eV. The former is assigned to nickel sulfide^[153]^ while the assignment of the latter peak is more difficult since it is out of the range of binding energy typically ascribed to S 2p. Since the feature at 157.5 eV is present only in the samples with Ni, it is most likely due to S bound to Ni, possibly in some different coordination site.

The high‐resolution C 1s, O 1s, S 2p, and Ru 2p XP spectra of the Ru‐loaded catalysts are shown in Figure 12. The Ru signal is very low, especially in the commercial supports. The C 1s spectra are quite similar to those recorded for Ni nanoparticles. The Ru 3 d doublet falls very close to the C 1s line, so that only the 3d_5/2_ line appears as a shoulder at 281 eV (inset of Figure 12). For this reason, the Ru 3p region was acquired as a fingerprint of Ru. Its inspection clearly demonstrates that Ru is present at the surface of the sample for Ru/Gnp and, in a lower amount, for Ru/rGO, while it is almost absent for the Ru/Com.Gnp sample. As for Ni‐loaded catalysts, we deduce that in the 0.5 %Ni/Gnp sample, Ru nanoparticles are distributed more at the surface and are hence more detectable.

The Ru 3p spectrum consists of a doublet (E_b_ for Ru 3p3/2 at 463.1 eV and Ru 3p 1/2at 485.3 eV) and its satellite doublet (E_b_ for Ru 3p3/2 at 466.6 eV and Ru 3p 1/2 at 489.0 eV).

These values do not fit with those expected for metallic Ru (3p3/2 at 461.2 eV with a small satellite at 470.9 eV) but are close to the values reported for RuO_2_, either anhydrous or hydrated.^[153]^ These two species are reported to have Ru 3p3/2 lines at 462.5 eV and 462.7 eV, respectively, with satellites at 465.4 and 465.6 eV respectively. They are therefore indistinguishable from our experimental resolution. The E_b_ for Ru 3d5/2 around 281 eV is coherent with this assignment.

The peaks at 485.3 and 489.0 eV correspond to the Ru 3p 1/2component of the Ru 3p doublet and do not provide any additional information.

The inspection of the O 1s spectra for Ru‐loaded samples enables to distinguish between anhydrous and hydrated RuO_2_. Indeed, a single peak at 531.8 eV is present for the Ru/Gnp sample, which upshifts to 532.7 eV on the commercial substrate. For anhydrous RuO_2_, the highest E_b_ component is expected at 529.3 eV with a lower intensity satellite at higher E_b_ while for the hydrated form, a broader peak is expected with a contribution at 530.9 eV.^[154]^ Ru/Gnp sample is thus likely to consist of hydrated RuO_2_. Since in the same work a contribution at 532.3 eV is assigned to O bonded to C, we believe this is the reason for the slightly higher E_b_ observed in our case and for the upshift detected for Ru/Com.Gnp, where a vanishing amount of Ru is detected. A possible alternative assignment is that the peak at 531.8 eV results from the overlap of a peak due to OH at 530.8 eV and one to H_2_O at 532.4 eV.^[155]^ Whatever the exact assignment, it is clear that XPS does not show any evident signature of the presence of metallic Ru and that RuO_2_ is at least partially hydrated.

For Ru/rGO, the O 1s spectrum shows a doublet at 531.1 eV and 533.6 eV, indicating the presence of hydrated RuO_2_ and water, respectively; the latter species was already present for the pure substrate.

As for the Ni‐based samples, peaks in the E_b_ range between 537 eV and 540 eV are present in the spectra. For both catalysts, we tentatively assign them to intercalated physiosorbed oxygen‐containing moieties such as O_2_ and H_2_O.[^156^, ^157^] Indeed, all other possible assignments of such high E_b_ species, as the presence of satellite lines and contamination by Sb or organic compounds, are ruled out in our experimental conditions.

The surveys in Figure S7 indicate the presence of minor N and S contaminants. High‐resolution S 2p shows a peak at ~161 eV. It is highest for the samples with a significant amount of Ru at the surface and is thus assigned to a ruthenium sulfide. S 2p lines around 162.1 eV have been reported.^[158]^ For the peak at ~158 eV, present only for Ru/rGO, the same consideration is reported above for the 157.5 eV peak hold. The Auger Ru MNN transition (not shown) is superimposed to the C KVV transition and does not provide any further hint.

XPS data confirmed the presence of oxidized/hydroxylated species that could either be produced by exposure to air of the samples or by slight oxidation in reaction conditions or can be due to physisorbed species. Moreover, the partial sulphidation of catalysts might hinder their reducibility in reacting conditions, accounting also for the applied pretreatment that was giving rise to oxidic species, before hydrogen introduction, in line with literature data. This is also in line with the poor hydrogenation activity observed when switching to H_2_/N_2_ mixture that produced small amounts of CH_4_ and no NH_3_, neither in transient conditions, indeed expected for Ru/C‐based materials. However, XP spectra have been collected after catalytic activity and thus the presence of CO_2_ and steam could contribute to this. The presence of oxidized Ni species has been evidenced in rWGS carried out over Ni(100).^[159]^ On the other hand, the surface enrichment with sulphur, could be responsible for the inhibition of the passive layer formation, thus allowing us to hypothesize that cationic species could be present also during catalytic activity.^[160]^

Thus, in excellent agreement with what was mentioned in the catalytic activity, partially oxidized Ni species appeared more active for rWGS, while sulphided Ru loss hydrogenation and hydrogenolysis activity when doped with sulphur for xylose hydrogenation (373 K, 4 MPa of hydrogen) and of glycerol hydrogenolysis,^[161]^ suggesting that S− might play a role in the stabilization of Ni and Ru in rWGS, accounting for an evaluated S− surface concentration of 0.5 wt %.

**Figure 13:**
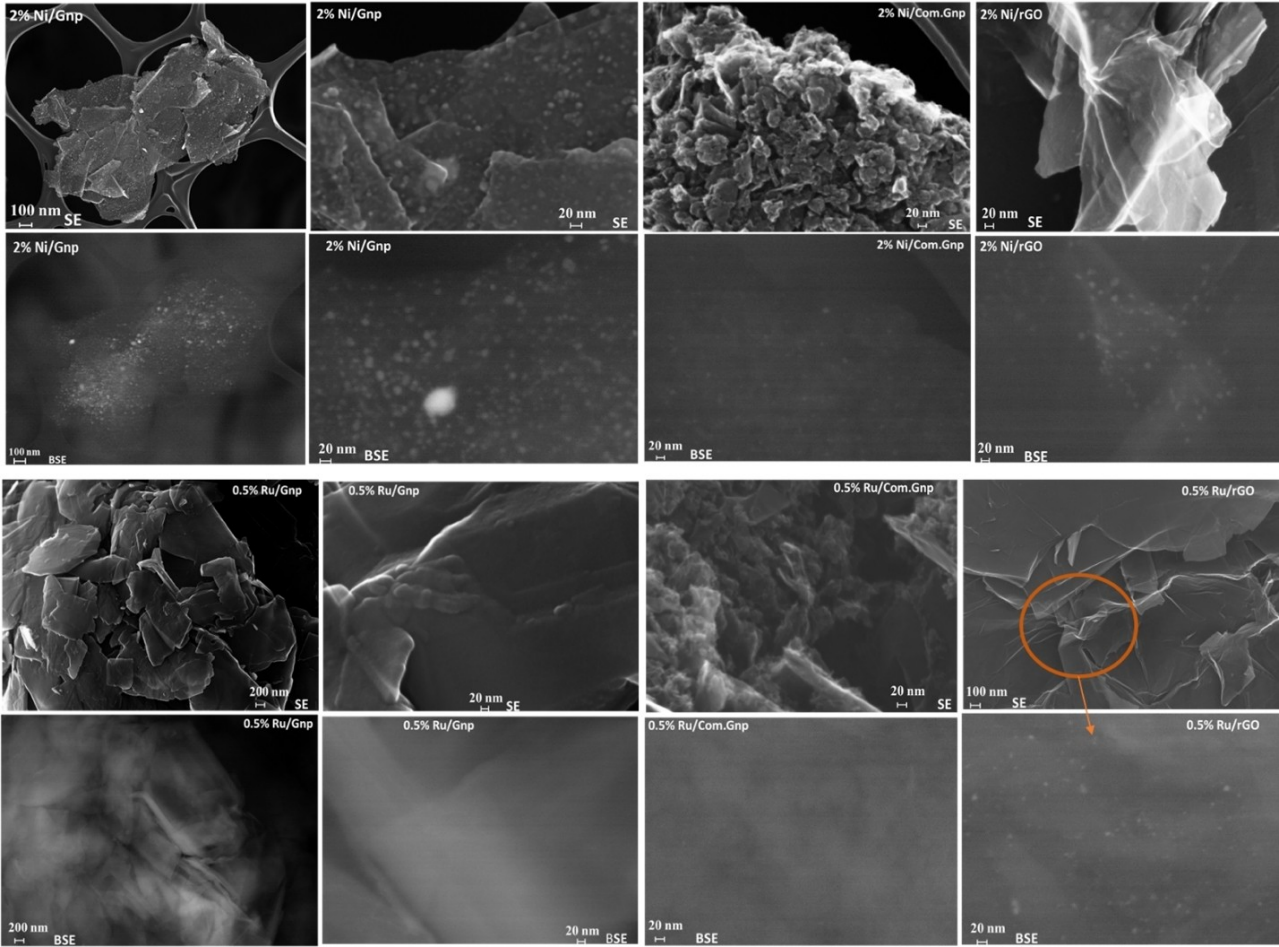
FE‐SEM images of spent catalysts (after catalytic activity). In each double row, the images on the bottom part display the backscattered electron signals of the corresponding image on the top, representing secondary electron signals. The first two columns from the left refer to GnP support, the third one to Com.Gnp, and the fourth one to rGO. The two topmost upper panels refer to Ni and the two lowermost ones to Ru.

FE‐SEM images of the exhausted catalysts, subjected to the hydrogenation test, are presented in Figure 13. Upon initial inspection and taking advantage of secondary electrons (SE), it is evident that Gnp‐, Com.Gnp‐, and rGO‐supported catalysts exhibit distinct morphological features. Gnp appears mostly as several graphitic sheets layered over each other, while Com.Gnp displays a 3D structure with wrinkles and grape‐like aggregates. Conversely, rGO can be observed as small pieces of folded and wrinkled graphitic layers.

In the Ni‐based series (upper double‐row), Ni nanoparticles are clearly visible with average particle sizes below 20 nm and homogeneously distributed on the support, by taking advantage of backscattered electrons. Additionally, in the case of Gnp support, it can be observed that some Ni nanoparticles, especially those grown at the edges of the C‐sheets, appear to be larger than those obtained in Com.Gnp and rGO, in line with the higher heterogeneity of the sample and the different degrees of exfoliation of the starting materials. Moreover, some brighter regions are also present where Ni is diffused through all the C‐layers, in line with the observations of XRD, Raman, and XPS. In the case of rGO, Ni nanoparticles were also spotted in the highly wrinkled regions of the support, confirming a certain intercalation in the rGO structure.

In Ru‐based series, 0.5 % Ru/rGO showed a morphology similar to the one of 2 % Ni/rGO, while for the others a diffused brightness at high magnification suggested the presence of dispersed Ru species, producing catalysts particularly performant and selective towards rWGS reaction, in line with literature data.^[150]^

**Figure 14:**
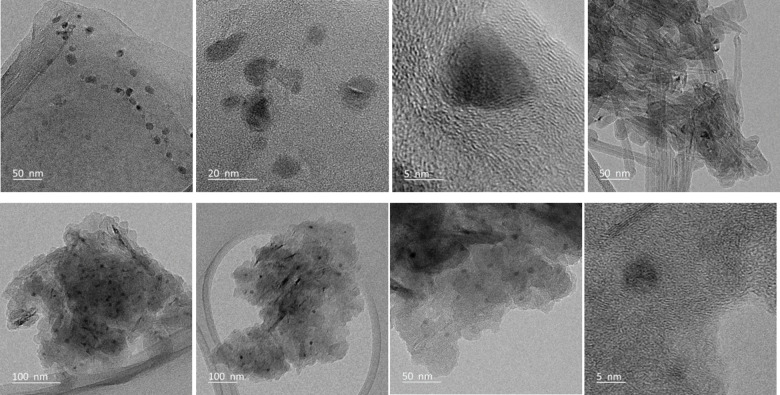
TEM images of spent 2 % Ni/rGO (upper row), and 2 % Ni/Com.Gnp (bottom row).

In the case of Ni/rGO and Ni/Com.Gnp, the TEM images (shown in Figure 14) confirm the FE‐SEM and XPS observations regarding the positioning of metal particles mainly in the outer boundary of C‐material, in‐between graphene layers, and displaying an average dimension of 5–15 nm. Many particles appear as well to be embedded in the material suggesting a certain confinement played by the different layers of rGO, confirming the metal intercalation. In the case of the Com.Gnp‐based, a more uniform distribution of the active phase particles seems to be present. Furthermore, TEM analysis revealed that Ni nanoparticles in 2 % Ni/Com.Gnp show also very small nanoparticles with an average size of ~5 nm (and lower), while in the case of 2 % Ni/rGO, the Ni particle sizes ranged from a minimum size of ~10 nm, ascribed in literature for most active particle size in the frame of CO_2_ hydrogenation to methane.[^5^, ^23^, ^143^] Thus, these data confirmed the possible role of oxidized for sulfided species in the high selectivity toward rWGS.

## 3 Conclusions

The main conclusion of the present work might be summarized as follows:

1. Homemade and commercial C‐based materials have been fully characterized and different impurities have been found, mainly connected to the followed preparation procedures i. e. Hummer′s method or to the use of chemicals upon preparation; a partial exfoliation of graphite has been successfully obtained for Gnp when using molten salts (NaCl‐KCl) in a modified synthetic procedure.
2. Carbon loss has been evaluated for all catalysts by applying a novel method that allowed to follow online using IR spectroscopy in the in‐situ thermal treatment before catalytic tests.
3. Ni− and Ru‐prepared catalysts resulted in being mainly active toward RWGS, even though, in the case of nickel, methane is observed among byproducts. Moreover, in the exhaust catalysts mainly cationic species have been found for tested materials together with the presence of S at the surface, interacting with C‐material and metals; this might play a role in the selectivity towards observed product, taking into account the concentration of S at the surface (0.5 wt %).
4. Best performances have been obtained for 0.5 %Ru over Gnp, both commercial and homemade, where the forecasted rWGS activity has been achieved at 723 K, thus being a promising candidate for CO_2_ conversion to CO. Instead, rGO‐based material appeared quite inactive in the whole temperature range. Apparent activation energies have been evaluated and confirmed the operation in the kinetic regime. For Ni/Gnp and Ni/Com.Gnp, the apparent activation energy is 90 kJ/mol while for Ru catalysts slightly different values are found i. e., 75 kJ/mol and 88 kJ/mol for Gnp and commercial Gnp, respectively.
5. An evaluation of the defect density (n_D_) and the distance of defects (L_D_) pointed out a quite different behavior for the investigated materials and suggested a slight decrease for Ru‐based catalysts and an opposite behavior for Ni ones.
6. Exhaust material showed a slight graphitization of the starting support and as well distributed Ru‐ and Ni‐based particles with a mean dimension of 5–10 and 10–25 nm, respectively.

## Conflict of Interests

The authors declare no conflict of interest.

4

## Supporting information

As a service to our authors and readers, this journal provides supporting information supplied by the authors. Such materials are peer reviewed and may be re‐organized for online delivery, but are not copy‐edited or typeset. Technical support issues arising from supporting information (other than missing files) should be addressed to the authors.

Supporting Information

## Data Availability

The data that support the findings of this study are available from the corresponding author upon reasonable request.
