# Supplementary material for: Graphene‐Based Material Supports for Ni− and Ru− Catalysts in CO2 Hydrogenation: Ruling out Performances and Impurity Role
Source: ChemSusChem. 2024 Oct 10;17(23):e202400993. doi: 10.1002/cssc.202400993 (PMC11632576; doi:10.1002/cssc.202400993)

ChemSusChem

Supporting Information

Graphene-Based Material Supports for Ni— and Ru—Catalysts in CO_2 Hydrogenation: Ruling out Performances and Impurity Role

Sina Ebrahim Atakoohi, Paola Riani, Elena Spennati, Letizia Savio, Luca Vattuone, Jacopo De Maron, and Gabriella Garbarino*

Graphene-based material supports for Ni- and Ru- catalysts in CO_2 Hydrogenation: ruling out performances and impurity role

Sina Ebrahim Atakoohi,[1] Paola Riani,[2,3] Elena Spennati,[1,3] Letizia Savio,[4] Luca Vattuone,[4,5] Jacopo De Maron[6], Gabriella Garbarino[1,3*]

[1]*Department of Civil, Chemical, and Environmental Engineering, University of Genova, Via Opera Pia 15, 16145 Genova, Italy*

[2]*Department of Chemistry and Industrial Chemistry, University of Genova, Via Dodecaneso 31, 16146 Genova, Italy*

[3]*INSTM, UdR Genova, Via Dodecaneso 31, 16146 Genova, Italy*

[4]*IMEM-CNR, Via Dodecaneso 33, 16146 Genova, Italy*

[5]*Department of Physics, University of Genova, Via Dodecaneso 33, 16146 Genova, Italy*

[6]*Department of Industrial Chemistry, University of Bologna, Viale del Risorgimento 4, 40126 Bologna, Italy*

e-mail: gabriella.garbarino@unige.it, phone +390103356029

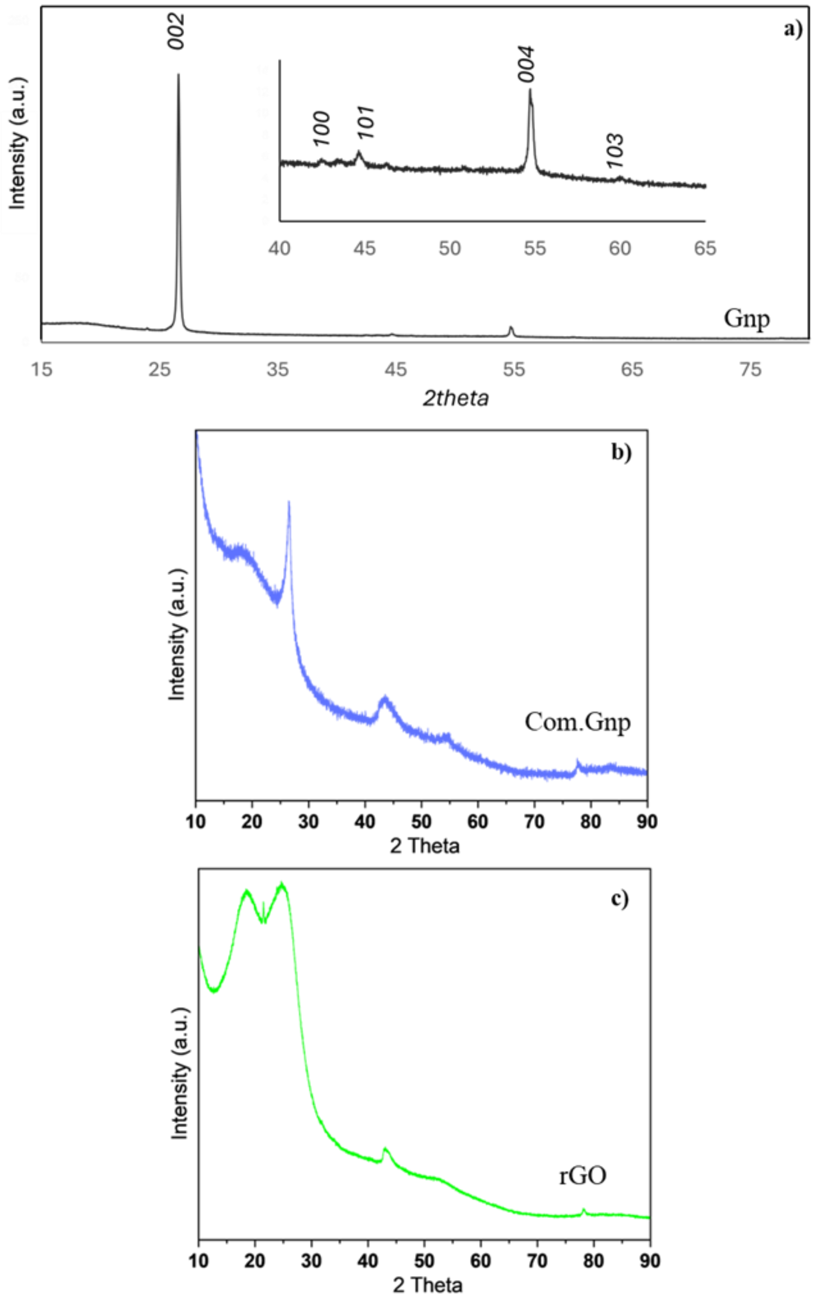

Figure S1. XRD of homemade GnP, Com.Gnp, and rGO in the 2theta range 10-90°.

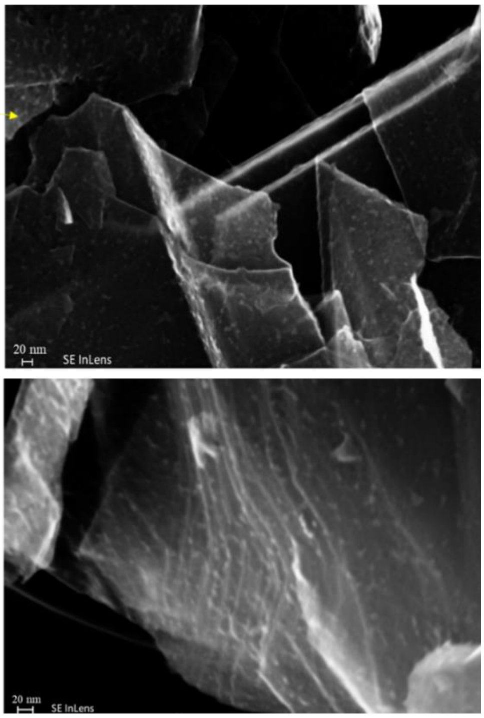

Figure S2. FE-SEM images of the lab-made Gnp synthesized by molten salt method at different magnifications by taking advantage of secondary electrons and an InLens detector.

Figure S3. SEM-EDXS elemental mapping for rGO support

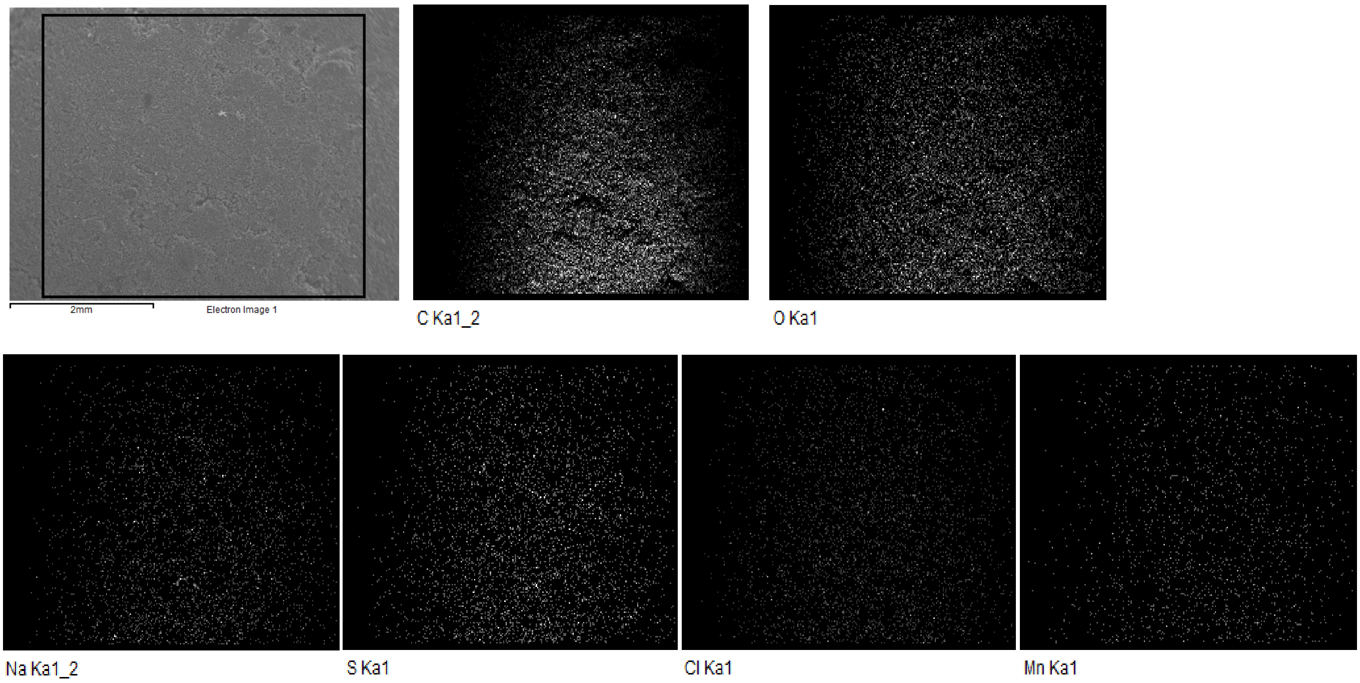

Figure S4. FT-IR spectra of Gnp, Com.Gnp and rGO based catalysts and supports range 2000-400 cm^{-1}. Gnp spectra are in offset scale while Com.Gnp and rGO are in common scale.

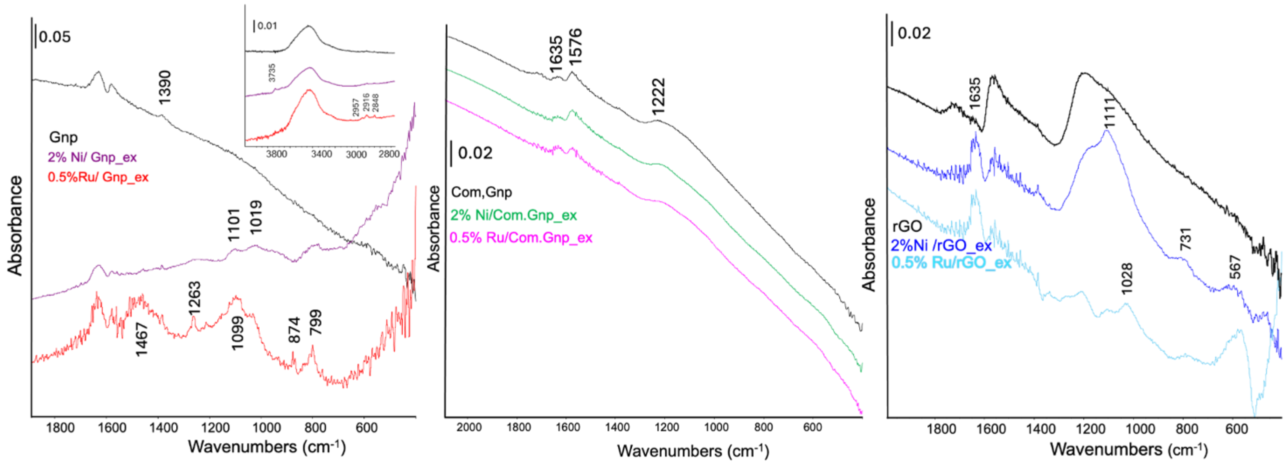

Table S1. XRD data analysis of exhausted catalysts: 2theta position and the evaluated d_{002}

Sample	2θ of 002 plane (°)	d_{002} (nm)
2%Ni/Gnp	26.56	0.336
2%Ni/Com.Gnp	26.57	0.335
2%Ni/rGO	26.04	0.342
0.5% Ru/Gnp	26.56	0.335
0.5%Ru/Com.Gnp	26.50	0.336
0.5% Ru/rGO	26.11	0.341

Figure S5. XPS overview spectra of the supports before particles loading.

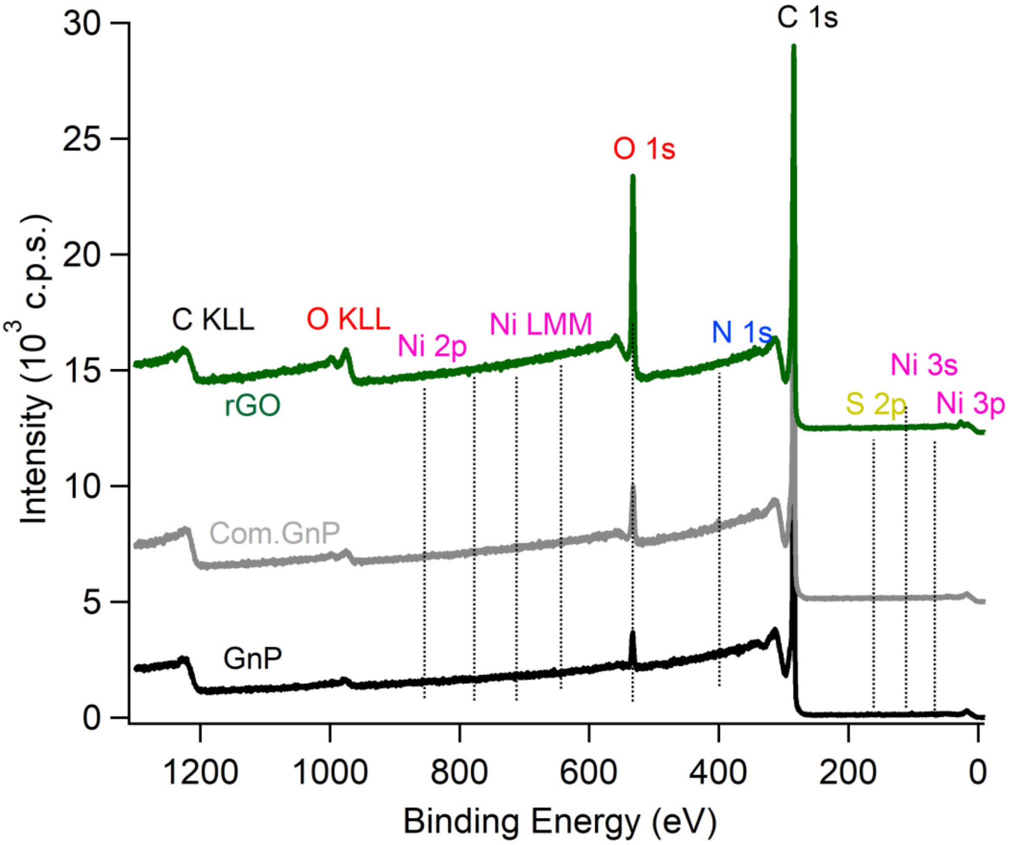

Figure S6. XPS overview of Ni-loaded samples after reaction.

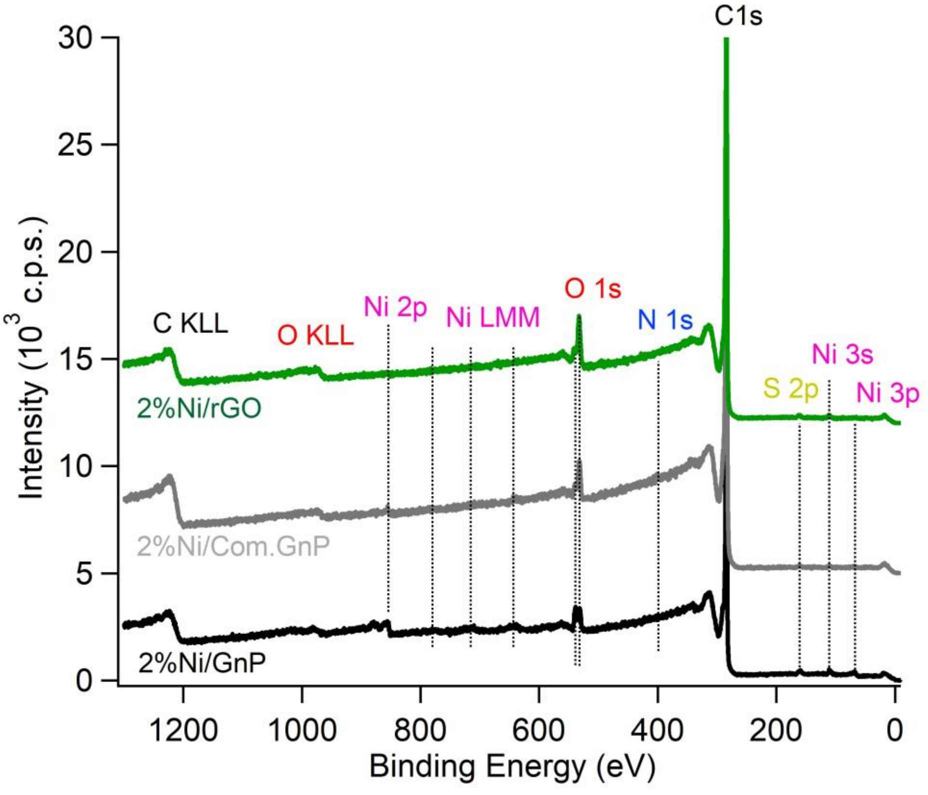

Figure S7. XPS overview spectra of Ru-loaded sample after reaction.

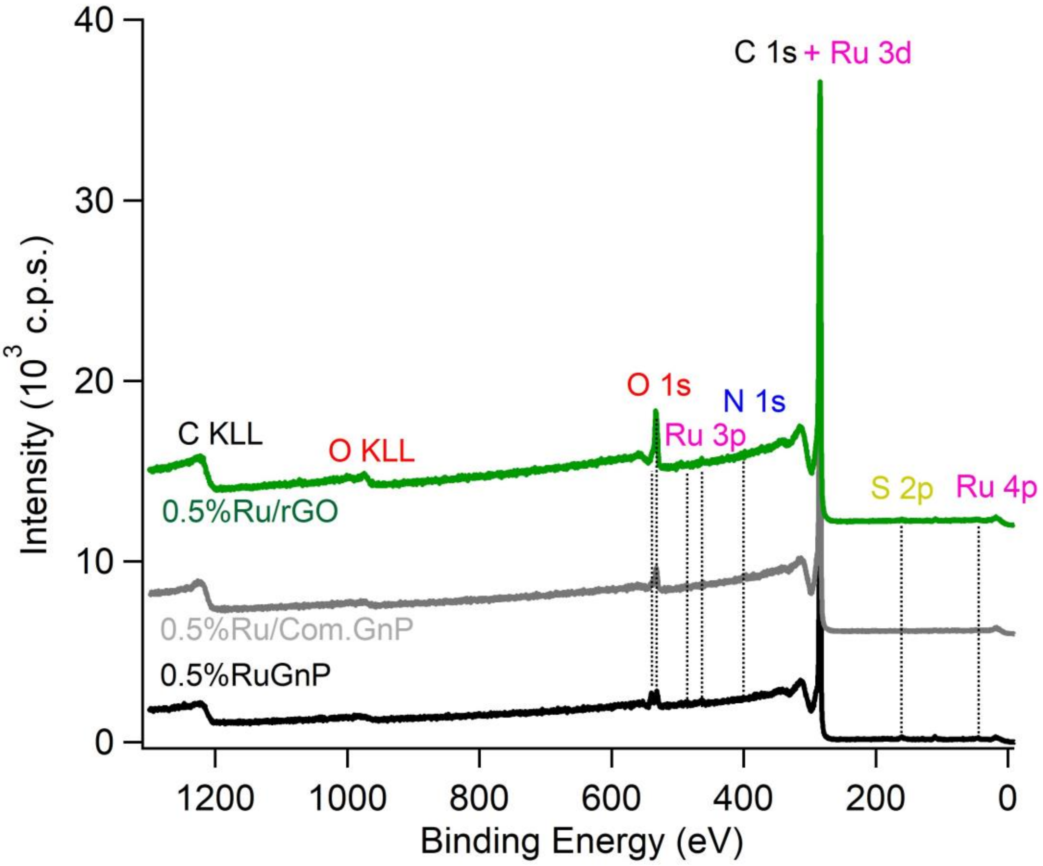

Figure S8. Auger Ni LMM spectra

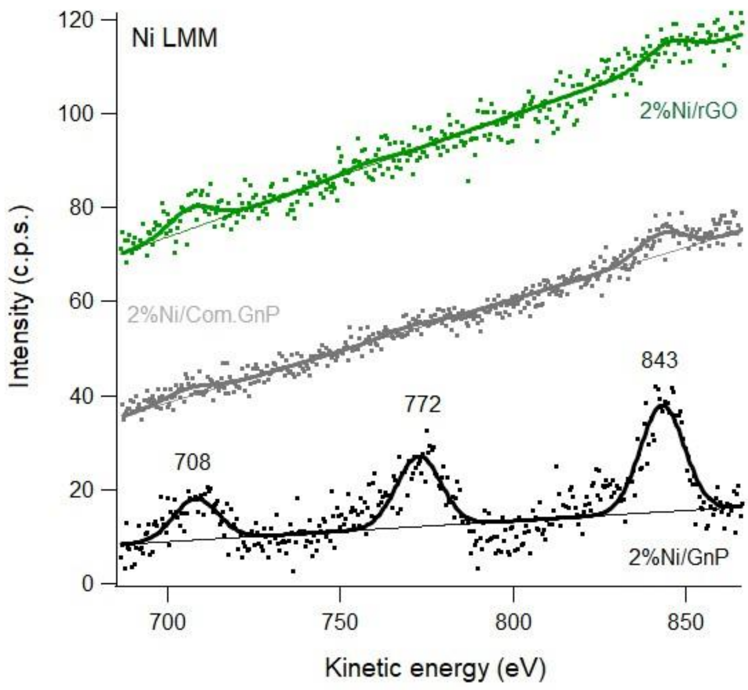

Supplement: Supplementary file 1 — Supporting Information [file CSSC-17-e202400993-s001.pdf]
